# *Paxillus involutus*-Facilitated Cd^2+^ Influx through Plasma Membrane Ca^2+^-Permeable Channels Is Stimulated by H_2_O_2_ and H^+^-ATPase in Ectomycorrhizal *Populus* × *canescens* under Cadmium Stress

**DOI:** 10.3389/fpls.2016.01975

**Published:** 2017-01-06

**Authors:** Yuhong Zhang, Gang Sa, Yinan Zhang, Zhimei Zhu, Shurong Deng, Jian Sun, Nianfei Li, Jing Li, Jun Yao, Nan Zhao, Rui Zhao, Xujun Ma, Andrea Polle, Shaoliang Chen

**Affiliations:** ^1^College of Biological Sciences and Technology, Beijing Forestry UniversityBeijing, China; ^2^College of Life Science, Jiangsu Normal UniversityXuzhou, China; ^3^School of Computer Science and Technology, Henan Polytechnic UniversityJiaozuo, China; ^4^Büsgen-Institut, Forstbotanik und Baumphysiologie, Georg-August-Universität GöttingenGöttingen, Germany

**Keywords:** ectomycorrhizal fungi, *Paxillus involutus*, MAJ, NAU, Cd^2+^-hyperaccumulator, poplar, NMT

## Abstract

Using a Non-invasive Micro-test Technique, flux profiles of Cd^2+^, Ca^2+^, and H^+^ were investigated in axenically grown cultures of two strains of *Paxillus involutus* (MAJ and NAU), ectomycorrhizae formed by these fungi with the woody Cd^2+^-hyperaccumulator, *Populus* × *canescens*, and non-mycorrhizal (NM) roots. The influx of Cd^2+^ increased in fungal mycelia, NM and ectomycorrhizal (EM) roots upon a 40-min shock, after short-term (ST, 24 h), or long-term (LT, 7 days) exposure to a hydroponic environment of 50 μM CdCl_2_. Cd^2+^ treatments (shock, ST, and LT) decreased Ca^2+^ influx in NM and EM roots but led to an enhanced influx of Ca^2+^ in axenically grown EM cultures of the two *P. involutus* isolates. The susceptibility of Cd^2+^ flux to typical Ca^2+^ channel blockers (LaCl_3_, GdCl_3_, verapamil, and TEA) in fungal mycelia and poplar roots indicated that the Cd^2+^ entry occurred mainly through Ca^2+^-permeable channels in the plasma membrane (PM). Cd^2+^ treatment resulted in H_2_O_2_ production. H_2_O_2_ exposure accelerated the entry of Cd^2+^ and Ca^2+^ in NM and EM roots. Cd^2+^ further stimulated H^+^ pumping activity benefiting NM and EM roots to maintain an acidic environment, which favored the entry of Cd^2+^ across the PM. A scavenger of reactive oxygen species, DMTU, and an inhibitor of PM H^+^-ATPase, orthovanadate, decreased Ca^2+^ and Cd^2+^ influx in NM and EM roots, suggesting that the entry of Cd^2+^ through Ca^2+^-permeable channels is stimulated by H_2_O_2_ and H^+^ pumps. Compared to NM roots, EM roots exhibited higher Cd^2+^-fluxes under shock, ST, and LT Cd^2+^ treatments. We conclude that ectomycorrhizal *P.* × *canescens* roots retained a pronounced H_2_O_2_ production and a high H^+^-pumping activity, which activated PM Ca^2+^ channels and thus facilitated a high influx of Cd^2+^ under Cd^2+^ stress.

## Introduction

The presence of highly toxic cadmium (Cd^2+^) in the environment is a serious threat to human health as heavy metals can be enriched in plants and eventually enter the human body through the food chain (Nawrot et al., 2006; Kaplan et al., 2011). The genus *Populus* spp. is of particular interest for phytoremediation of Cd^2+^ pollution (Sell et al., 2005; Krpata et al., 2008, 2009; Kieffer et al., 2009; He et al., 2011, 2013, 2015; Ma Y. et al., 2014), due to its widespread distribution, rapid growth, and genotypic differences in response to ion-specific stress (Chen and Polle, 2010; Polle et al., 2013; Chen et al., 2014; Polle and Chen, 2015). *Populus tremula* (Kieffer et al., 2009) and *Populus* × *canescens* (He et al., 2011) have been recently identified as woody Cd^2+^-hyperaccumulators. Cd^2+^ enrichment in these poplars (Kieffer et al., 2009; He et al., 2011; Ma Y. et al., 2014) exceed the threshold of 100 μg Cd^2+^ g^-1^ DW that has commonly been defined for hyperaccumulation (Milner and Kochian, 2008; Krämer, 2010). He et al. (2013) demonstrated that *P.* × *canescens* could detoxify Cd^2+^ by its sequestration in the bark.

In nature, poplar roots form symbioses with mycorrhizal fungi (Danielsen et al., 2012, 2013). For example, colonization of *P.* × *canescens* roots with the ectomycorrhizal fungus *Paxillus involutus* improves growth, primes for increased stress tolerance, increases nutrition, and regulates the ion balance under salt stress (Schützendübel and Polle, 2002; Gafur et al., 2004; Langenfeld-Heyser et al., 2007; Luo et al., 2009, 2011; Li J. et al., 2012; Ma X. et al., 2014). A notable finding was that *Paxillus involutus* ectomycorrhizas enhance both Cd^2+^ uptake and tolerance in *P.* × *canescens* (Ma Y. et al., 2014). Thus, ectomycorrhizal poplar plants offer a great potential for phytoremediation of Cd^2+^-polluted soils (Sell et al., 2005; Krpata et al., 2008, 2009; Luo et al., 2014; Ma Y. et al., 2014).

Cd^2+^ is generally believed to enter plant cells through high affinity transporters responsible for the uptake of divalent cations (Cu^2+^, Co^2+^, Fe^2+^, Ca^2+^, Mn^2+^, and Zn^2+^; Liu et al., 1997; Clemens et al., 1998; Cohen et al., 1998; Hirschi et al., 2000; Thomine et al., 2000; Zhao et al., 2002; Cosio et al., 2004; Clemens, 2006; Roth et al., 2006). Cd^2+^ can even induce nutrient deficiencies by competing with the uptake of essential elements (Zhao et al., 2006; Papoyan et al., 2007; DalCorso et al., 2008; Gallego et al., 2012; Baliardini et al., 2015). On the other hand, elevated Ca^2+^ levels suppress Cd^2+^ uptake in different ecotypes of *Sedum alfredii* also supporting competition of Cd^2+^ uptake with nutrient cations (Lu et al., 2010). Transcript levels of the transporters involved in Cd^2+^ uptake and transport have been investigated in herbaceous and woody species (Kim et al., 2006; Plaza et al., 2007; Krämer, 2010; Migeon et al., 2010; Mendoza-Cózatl et al., 2011; Lin and Aarts, 2012). In poplar plants, a variety of heavy metal transporters, such as ZRT-IRT-like proteins (ZIP2, ZIP6.2), natural resistance associated macrophage proteins (NRAMP1.1, NRAMP1.3), ATP-binding cassette transporter C1 (ABCC1), heavy metal ATPase 4 (HMA4), ATP-binding cassette transporter in mitochondria (ATM3), have been suggested to play pivotal roles in Cd^2+^ transport and detoxification (Ma Y. et al., 2014; He et al., 2015). In addition to these heavy metal transporters, ion channels in the plasma membrane (PM) that are permeable to Cd^2+^ contribute the Cd^2+^ uptake (Li et al., 2012a; Sun et al., 2013a,b; He et al., 2015). High external Cd^2+^ concentrations establish a large electrochemical gradient facilitating the rapid movement of Cd^2+^ ions through Cd^2+^-permeable channels. Perfus-Barbeoch et al. (2002) suggested that Cd^2+^ enters root cells via plasma membrane (PM) Ca^2+^ channels.

Ca^2+^ channels in the PM have been characterized by electrophysiological measurements involving incorporation of plasma-membrane vesicles into planar lipid bilayers (PLB, White, 2000) and patch clamping (Perfus-Barbeoch et al., 2002). According to their electrophysiological properties, the channels can be divided into depolarisation-, hyperpolarisation-, elicitor-activated, and voltage-insensitive channels (Thuleau et al., 1998; White, 2000). These channels display different sensitivities to typical inhibitors of Ca^2+^ channels, such as La^3+^, Gd^3+^, TEA, and verapamil. Specifically, verapamil and TEA inhibit depolarisation-activated Ca^2+^ channels, such as the wheat root channel rca (Piñeros and Tester, 1997; White, 1998), and rye root voltage-dependent cation channel 2, VDCC2 (White, 1998). La^3+^ shares a high similarity to another trivalent cation, Gd^3+^. Both cations are able to inhibit three distinct classes of Ca^2+^ channels, including depolarisation-activated Ca^2+^ channels, rca (Piñeros and Tester, 1997; White, 1998), hyperpolarisation-activated Ca^2+^ channels (HACCs) in onion bulb epidermis (Pickard and Ding, 1993), voltage-insensitive channels such as Arabidopsis root epidermal non-selective cation channels (NSCCs; Demidchik et al., 2002), and large-conductance elicitor-activated channel (LEAC) in parsley cell suspension (Zimmermann et al., 1997). Ca^2+^ channels in the PM are permeable to divalent (including Ca^2+^, Mg^2+^, Ba^2+^, Sr^2+^, Co^2+^, Zn^2+^, Mn^2+^, Ni^2+^, Cu^2+^; Cosgrove and Hedrich, 1991; Ping et al., 1992; Pickard and Ding, 1993; Thuleau et al., 1994a,b; Gelli and Blumwald, 1997; Zimmermann et al., 1997; White, 1998; Grabov and Blatt, 1998, 1999) and monovalent cations (Na^+^, K^+^, Cs^+^, Li^+^, Rb^+^; Cosgrove and Hedrich, 1991; Pickard and Ding, 1993; Zimmermann et al., 1997; Piñeros and Tester, 1997; White, 1998). In accordance with the suggestion that Cd^2+^ ions can be transported into cells through Ca^2+^ channels (Perfus-Barbeoch et al., 2002; Gallego et al., 2012; Li et al., 2012b) the permeability for Cd^2+^ through wheat VDCC2 was detected when the plasma membrane derived from root cells was incorporated into PLB (White, 1998). Using the whole-cell patch-clamp technique, Perfus-Barbeoch et al. (2002) confirmed that Cd^2+^ permeates through the PM Ca^2+^ channels in Arabidopsis guard cells. The Cd^2+^ influx was effectively blocked by Ca^2+^ channel blockers, e.g., LaCl_3_ and verapamil in *Suaeda salsa* (Li et al., 2012a), *Populus euphratica* (Sun et al., 2013b), and *P. tremula* × *P. alba* (He et al., 2015), further indicating that Cd^2+^ ions penetrate into plant cells through Ca^2+^-permeable channels.

It is possible that hydrogen peroxide (H_2_O_2_) stimulates the entry of Cd^2+^ through PM Ca^2+^ channels as the activity of these channels has been shown to be stimulated by H_2_O_2_. Pei et al. (2000) found that H_2_O_2_ activates the PM Ca^2+^ channels, leading to a subsequent rise of cytosolic Ca^2+^ in *Arabidopsis* guard cells. Demidchik et al. (2007) observed a transient increase of Ca^2+^ influx in the root epidermis when exogenous H_2_O_2_ was applied to *Arabidopsis thaliana*. In NaCl-stressed *P. euphratica* cells, Ca^2+^ influx through Ca^2+^ channels was activated by H_2_O_2_ (Sun et al., 2010). Recently, H_2_O_2_ was shown to accelerate Cd^2+^ influx in *P. euphratica* cells, while the H_2_O_2_-stimulated Cd^2+^ influx was blocked by LaCl_3_ (Sun et al., 2013b; Han et al., 2016). Moreover, the application of a H_2_O_2_ scavenger, catalase, lowered the Cd^2+^ influx across the PM in Cd^2+^-stressed *P. euphratica* cells (Sun et al., 2013b). In Cd^2+^-treated *P. euphratica* cells, hydrogen sulfide was found to reduce Cd^2+^ influx through down-regulation of H_2_O_2_-stimulated Cd^2+^ transport across the PM Ca^2+^ channels (Sun et al., 2013b). H_2_O_2_ is not only produced in Cd^2+^-stressed poplar cells (Sun et al., 2013b; Han et al., 2016) and roots (Ma Y. et al., 2014; He et al., 2015), but is also massively enriched in *Populus* × *canescens*–*Paxillus involutus* ectomycorrhizal associations (Gafur et al., 2004; Langenfeld-Heyser et al., 2007). Thus, it can be speculated that the fungal-elicited H_2_O_2_ accelerates the entry of Cd^2+^ through PM Ca^2+^ channels. However, this hypothesis needs to be clarified by further electrophysiological investigations.

In addition to H_2_O_2_, the PM H^+^-ATPase plays a crucial role in accelerating Cd^2+^ transport in poplar roots (Ma Y. et al., 2014; He et al., 2015). He et al. (2015) demonstrated that the net Cd^2+^ influx was pH-dependent in poplar roots and effectively blocked by inhibitors of H^+^-pumps. Ma Y. et al. (2014) showed that the active PM H^+^-ATPase-driven Cd^2+^ uptake is a major factor for increased Cd^2+^ accumulation in ectomycorrhizal (EM) poplar plants. They suggested that the EM-induced transcripts of *HA2.1* and *AHA10.1* genes, encoding PM H^+^-ATPases in *P*. × *canescens*, may result in H^+^-pump-stimulated Cd^2+^ enrichment (Ma Y. et al., 2014). In agreement with this suggestion transgenic poplars that were more Cd^2+^ tolerant by overexpression of γ-glutamylcysteine synthetase, showed upregulated transcript levels of *VHA1.1*, *HA2.1* and *AHA10.1* and a high Cd^2+^ uptake rate (He et al., 2015). The PM H^+^-ATPases maintain a H^+^ gradient across the membrane to promote active transport of essential elements across the PM (Beritognolo et al., 2007; Ma et al., 2010; Sun et al., 2010; Luo et al., 2013). Increased H^+^-pumping activities have been well characterized in arbuscular mycorrhizal associations (Ramos et al., 2005; Rosewarne et al., 2007) and in ectomycorrhizal associations formed by *Paxillus involutus* (strains MAJ and NAU) with *Populus* × *canescens* (Li J. et al., 2012). We have previously shown that the upregulated H^+^-pumping activities in *Paxillus involutus*-*Populus* × *canescens* symbiosis resulted in enhanced Ca^2+^ uptake and enrichment (Li J. et al., 2012). Demidchik et al. (2002) proposed that voltage modulation of the co-existing NSCC/HACC by PM H^+^-ATPase would be a potent regulator for Ca^2+^ entry to the root cell cytoplasm. The high H^+^-pumping activity leads to hyperpolarization of the PM and, thus, may increase Cd^2+^ influx through hyperpolarisation-activated Ca^2+^ channels. However, it is unknown whether the PM H^+^-ATPases could stimulate the entry of Cd^2+^ through Ca^2+^-permeable channels in ectomycorrhizal plants.

The two *P. involutus* strains, MAJ and NAU, form different colonization structures with *P.* × *canescens* roots (Gafur et al., 2004). Strain MAJ forms a typical hyphal mantle and Hartig net with roots of *P.* × *canescens*, while NAU is unable to intrude between the host cells and forms only a hyphal mantle ensheathing the root tips (Gafur et al., 2004). The colonization of *P.* × *canescens* roots with the competent strain MAJ results in enriched Cd^2+^ levels under Cd^2+^ stress (Ma Y. et al., 2014). Whether the incompatible fungal isolate NAU also affects the Cd^2+^ entry into *P.* × *canescens* host plants needs to be clarified.

In this study, we used a non-invasive micro-test technique (NMT) to measure fluxes of Cd^2+^, Ca^2+^ and H^+^ in Cd^2+^-stressed roots of non-mycorrhizal (NM) and ectomycorrhizal *P.* × *canescens* plants colonized with *Paxillus involutus* strains, MAJ and NAU. The aim was to elucidate whether the Cd^2+^ influx through Ca^2+^-permeable channels is stimulated by H_2_O_2_ and H^+^-ATPase in ectomycorrhizal roots since the ectomycorrhizas exhibit enhanced H_2_O_2_ production and upregulated H^+^-pumping activity. NMT microelectrodes measure the ion fluxes on the surface of the tissues, which are either the plant root cells for the NM plants or the fungal hyphae forming the mantle structure ensheathing the roots. To discriminate between potentially different Cd^2+^ effects on fungus and plant roots, fluxes of Cd^2+^, Ca^2+^ and H^+^ were examined for pure fungal mycelia of the two *P. involutus* isolates, MAJ and NAU, in addition to flux recordings on NM and EM roots. Furthermore, flux profiles of Cd^2+^ and Ca^2+^ were recorded in *P. involutus*-inoculated roots after 7 days of co-culture. The aim was to determine whether flux profiles of mature EM associations resemble the pattern of those from host roots at early stages of fungal colonization when the host is known to activate transient defense responses in contrast to the mature ectomycorrhizal symbioses (Duplessis et al., 2005).

## Materials and Methods

### Fungus and Plant Cultures for EM Colonization

The *Paxillus involutus* isolates MAJ and NAU, obtained from the Büsgen Institute: Institute of Forest Botany and Tree Physiology (Göttingen University, Germany), were grown on 2% modified Melin Norkrans (MMN) agar medium (g⋅L^-1^): KH_2_PO_4_ 0.5, (NH_4_)_2_SO_4_ 0.25, MgSO_4_⋅7H_2_O 0.15, CaCl_2_⋅2H_2_O 0.05, NaCl 0.025, FeCl_3_⋅6H_2_O 0.01, thiamine HCl 0.0001, glucose 10, malt extract 3, pH 5.2 (Gafur et al., 2004; Li J. et al., 2012). Prior to the colonization, the fungi were pre-grown on the agar culture medium for 1 week in petri dishes (diameter 90 mm) and kept in darkness at 23°C.

Plantlets of *Populus* × *canescens* (a hybrid of *Populus tremula* × *Populus alba*) were propagated by micropropagation as described by Leple et al. (1992). Regenerated *P.* × *canescens* plants were grown for 3–4 weeks on Murashige and Skoog (MS) medium (Murashige and Skoog, 1962). Uniform plants with sufficient roots were used for ectomycorrhization. The colonization of *P.* × *canescens* with *Paxillus involutus* strains MAJ and NAU was followed the procedures described by Gafur et al. (2004). In brief, rooted plantlets from sterile culture were placed on the MMN agar medium in the presence or absence of EM mycelium. After fungal inoculation, the petri dishes were sealed with Parafilm and covered with aluminum foil to keep the roots in darkness. During the period of incubation, the temperature in the climate chamber was maintained at 23°C with a light period of 16 h (6:00 AM–22:00 PM). Photosynthetic active radiation (PAR) of 200 μmol m^-2^ s^-1^ was supplied by cool white fluorescent lamps. After 1 month of inoculation, EM and NM root tips for anatomical investigations were embedded, stained, and photographed as described previously (Gafur et al., 2004). EM and NM plants with similar height and growth performance were used for CdCl_2_ treatment.

### Liquid Culture of Fungi

Liquid culture of *P. involutus* was grown as previously described (Ott et al., 2002; Langenfeld-Heyser et al., 2007; Li J. et al., 2012). In brief, mycelium from the agar plate was homogenized, transferred into 100 mL of liquid medium (pH 4.8) in flasks, and incubated on a rotary shaker in darkness (150 rpm, 23°C). *P. involutus* in submerged culture grew in the form of compact spherical masses of mycelium (pellets). For Cd^2+^ shock treatment, sterile filtered CdCl_2_ solutions were added to achieve final concentrations of 50 μM. After ST (24 h) or LT (7 days) treatment, axenic cultures of MAJ and NAU were used for steady flux measurements of Cd^2+^, H^+^, and Ca^2+^.

### Cadmium Treatment

Ectomycorrhizal and non-mycorrhizal plants were carefully removed from MMN agar medium. Rooted plantlets were cultivated in individual pots containing hydroponic MS nutrient solution (MS medium without agar and sucrose) (Murashige and Skoog, 1962). Plants were covered with plastic bags to reduce the rapid water loss in a growth room. NM and EM plantlets were subjected to 50 μM CdCl_2_ for a short-term (ST) exposure, 24 h or a long-term (LT) exposure for 7 days. The required amount of CdCl_2_ was added to the MS nutrient solution. Control plants were treated in the same manner without the addition of CdCl_2_. The plants were maintained at 23°C with a light period of 16 h (6:00 AM–22:00 PM) and PAR was 200 μmol m^-2^ s^-1^. Plants were continuously aerated by passing air to hydroponic MS nutrient solution, which was regularly renewed. Steady fluxes of Cd^2+^, Ca^2+^ and H^+^ in NM and EM roots were examined after 24 h and 7 days of CdCl_2_ treatment. In addition, ST-induced alterations of Cd^2+^ and Ca^2+^ fluxes were also examined in non-inoculated and *P. involutus*-inoculated roots after 7 days of co-culture.

### Measurements of Net Cd^2+^, Ca^2+^, and H^+^ Fluxes

#### Preparations of Ion-Selective Microelectrodes

Non-invasive Micro-test Technique (NMT-YG-100, Younger USA LLC, Amherst, MA01002, USA) with ASET 2.0 (Sciencewares, Falmouth, MA 02540, USA) and iFluxes 1.0 Software (Younger USA, LLC, Amherst, MA 01002, USA) was used to monitor fluxes of Cd^2+^, Ca^2+^ and H^+^ in EM and NM roots (Sun et al., 2009a,b; Sun et al., 2013a,b; Ma X. et al., 2014). Ion-selective electrodes were prepared as described in Sun et al. (2009a, 2013a) and Ma X. et al. (2014). Briefly, pre-pulled and silanized glass micropipettes (diameter 4–5 μm, XY-DJ-01; Xuyue (Beijing) Science and Technology Co. Ltd., Beijing, China) were back-filled with backfilling solution [Cd^2+^ microelectrodes: 10 mM Cd(NO_3_)_2_ and 0.1 mM KCl; Ca^2+^ microelectrodes: 100 mM CaCl_2_; H^+^ microelectrodes: 40 mM KH_2_PO_4_ and 15 mM NaCl, pH 7.0] to a length of 1.0 cm from the tip. Then the micropipettes were front-filled with 15 μm columns of selective liquid ion exchange cocktails (LIXs) (Cd: Fluka 20909, Sigma–Aldrich, St Louis, MO, USA; Ca: Fluka 21048; H: Fluka 95293 Fluka Chemie GmbH, Buchs, Switzerland). An Ag/AgCl wire electrode holder (XYEH01-1; Xuyue Sci. and Tech. Co., Ltd.) was inserted in the back of the electrode to create an electrical contact with the electrolyte solution. DRIREF-2 (World Precision Instruments, Inc., Sarasota, FL, USA) was used as the reference electrode (CMC-4). Prior to the measurements, ion-selective microelectrodes for the target ions were calibrated by the following standard solution:

(1) Cd^2+^: 0.01, 0.05, 0.1 mM (Cd^2+^ concentration was 0.05 mM in the measuring solution);
(2) Ca^2+^: 0.1, 0.5, 1.0 mM (Ca^2+^ was 0.2 mM in the measuring buffer);
(3) H^+^: pH 4.2, 5.2, 6.2 (pH of the measuring solution was adjusted to 5.2 with KOH and HCl for root samples).

Electrodes were used when the Nernstian slopes in ranges of 29 ± 3 mV/decade (Cd^2+^, Ca^2+^) and 58 ± 5 mV/decade (H^+^). The flux rate was calculated on the basis of Fick’s law of diffusion:

*J* = -*D* (*dc*/*dx*),

where *J* is the ion flux in the *x* direction, *D* is the ion diffusion coefficient in a particular medium, *dc* represents the ion concentration difference, *dx* is the microelectrode movement between two positions, and *dc/dx* represents the ion concentration gradient. As part of the NMT system, ASET software [Science Wares (East Falmouth, MA, USA) and Applicable Electronics], was used for data and image acquisition, preliminary processing, control of three-dimensional electrode positioner and stepper-motor-controlled fine focus of the microscope stage.

#### Experimental Protocols for Steady-State Flux Measurements

Cd^2+^, Ca^2+^, and H^+^ fluxes were non-invasively measured by moving the ion-selective microelectrode between two positions close to the materials in a preset excursion (30 μm for excised roots and fungal mycelia) at a programmable frequency in the range of 0.3–0.5 Hz. *P. involutus* mycelia, EM and NM roots from the ST and LT CdCl_2_ treatments were rinsed with re-distilled water for 2–3 times, and then incubated in the basic measuring solution to equilibrate for 25 min. The concentration gradients of Cd^2+^, Ca^2+^, and H^+^ were measured as previously described (Li J. et al., 2012; Lu et al., 2013; Sun et al., 2013a,b).

(1) Cd^2+^ measuring solutions: 0.1 mM KCl, 0.1 mM MgCl_2_, 0.05 mM CaCl_2_ and 0.05 mM CdCl_2,_ pH was adjusted to 5.2 with KOH and HCl;
(2) Ca^2+^ measuring solutions: 0.1 mM NaCl, 0.1 mM MgCl_2_, 0.1 mM KCl, and 0.2 mM CaCl_2_, pH was adjusted to 5.2 with KOH and HCl;
(3) H^+^ measuring solutions: 0.1 mM NaCl, 0.1 mM MgCl_2_, 0.1 mM CaCl_2_ and 0.5 mM KCl, pH 5.2 was adjusted with KOH and HCl.

The steady fluxes of roots were then recorded 100 μm from the apex and conducted along the root axis until 2300 μm at intervals of 200–300 μm. The fluxes of each measuring point in apical regions were continuously recorded for 6–8 min. For *P. involutus* mycelia, Cd^2+^, Ca^2+^, and H^+^ fluxes were measured around the surface of pelleted hyphae over a recording period of 30 min.

#### Transient Flux Recording

*Paxillus involutus* fungal mycelia and roots sampled from EM and NM plants were immobilized in the measuring solutions of Cd^2+^ (0.1 mM KCl, 0.1 mM MgCl_2_, 0.05 mM CaCl_2_, pH 5.2); Ca^2+^ (0.1 mM NaCl, 0.1 mM MgCl_2_, 0.1 mM KCl, and 0.2 mM CaCl_2_, pH 5.2) and H^+^ (0.1 mM NaCl, 0.1 mM MgCl_2_, 0.1 mM CaCl_2_ and 0.5 mM KCl, pH 5.2) for 25 min equilibration. Then the steady-state fluxes in fungal mycelia and the root apical region (100 μm from the root apex) were continuously recorded for 5 min prior to the CdCl_2_ shock. CdCl_2_ stock (100 μM) was slowly added to the measuring solution using a pipette until the final Cd^2+^ concentration reached 50 μM. Afterward, transient kinetics of Cd^2+^, Ca^2+^, and H^+^ were restarted and continued for 40 min. The data measured during the first 1–2 min was discarded, due to the effects of the diffusing stock solution. The high flux of Cd^2+^, Ca^2+^, and H^+^ during the following 2 min was defined as peaking values.

Effects of H_2_O_2_ on CdCl_2_-altered transient kinetics of Cd^2+^ and Ca^2+^ were also examined in NM and EM roots. Following the CdCl_2_ shock (50 μM) as described above, H_2_O_2_ (1.0 mM) was introduced to the measuring solution and transient kinetics of Cd^2+^ and Ca^2+^ were recorded for 20 min.

Fungal mycelia were exposed to 50 μM CdCl_2_ to induce a shock. Cd^2+^, Ca^2+^, and H^+^ fluxes were monitored over a continuous recording period of 40 min. For transient flux kinetics, the data measured during the first 1–2 min were discarded due to the diffusion effects of stock addition.

### Effects of Ca^2+^ on Sensitivity of Cd^2+^ Electrodes

To determine whether Ca^2+^ ions compete with Cd^2+^ to penetrate across PM Ca^2+^-permeable channels, the effects of additional Ca^2+^ ions on Cd^2+^ electrodes was examined. Cd^2+^ calibrating solutions were added with 0, 0.01, 0.025, 0.05, 0.1, 0.2, 0.5, 1.0, or 2.0 mM Ca^2+^. Then Cd^2+^ microelectrodes were calibrated in Ca^2+^-supplemented solutions as described above. Moreover, the Nernst slope and intercept of the Cd^2+^ electrodes were calibrated in the measuring solution containing 0.1 mM KCl, 0.1 mM MgCl_2_, and 0.05 mM CaCl_2_.

### Flux Oscillations

Oscillations in membrane-transport activity are ubiquitous in plant response to salinity, temperature, osmotic, hypoxia, and pH stresses (Shabala et al., 2006). In our study, rhythmic (ultradian) flux oscillations in NM and EM *P.* × *canescens* roots were not noticeable as that observed in herbaceous species (Shabala et al., 1997, 2003, 2006; Shabala and Knowles, 2002). This finding is presumably due to a lower growth rate of woody roots compared with crop species (Li J. et al., 2012). The flux oscillations of the measured ions, e.g., H^+^, Ca^2+^, and Cd^2+^, were more like fluctuations as previously reported in poplar roots (e.g., Na^+^, K^+^, H^+^, and Ca^2+^; Li J. et al., 2012). In this study, H^+^, Ca^2+^, and Cd^2+^ fluxes were recorded for 6–8 min at each point, which is long enough to cover oscillatory periods of measured ions.

### Inhibitor and Stimulator Treatment

In this study, the effects of Ca^2+^, pH, H_2_O_2_, and PM transporter and channel inhibitors on Cd^2+^-altered ion flux profiles were examined in fungal mycelia and roots (NM and EM). Briefly,

*Series 1: Ca^2+^ channel inhibitors*. NM and EM roots were pre-treated with or without LaCl_3_ (5 mM; Sun et al., 2010; Li et al., 2012b), GdCl_3_ (500 μM, Demidchik et al., 2007, 2009; Sun et al., 2012), TEA (50 μM, White, 1998; Li J. et al., 2012), or verapamil (20 μM, Li et al., 2012a; He et al., 2015) for 24 h in the presence and absence of 50 μM CdCl_2_. Fungal mycelia of the two *P. involutus* isolates, MAJ and NAU, were subjected to 0 or 5 mM LaCl_3_ treatment for 24 h supplemented with or without 50 μM CdCl_2_.

*Series 2: Ca^2+^*. After being subjected to Cd^2+^ stress (CdCl_2_, 50 μM) for 24 h, NM and EM roots were then exposed to 25, 50, or 100 μM CaCl_2_ for flux recordings in the presence of CdCl_2_.

Cd^2+^ and Ca^2+^ fluxes in Series 1 and 2 were measured along root axes, 100–2,300 μm from the apex, at intervals of 200–300 μm. In *P. involutus* mycelia, Cd^2+^ and Ca^2+^ fluxes were continuously measured around the surface of pelleted hyphae over a recording period of 30 min.

*Series 3: Hydrogen peroxide*. NM and EM roots were sampled and immobilized in Cd^2+^ or Ca^2+^ measuring solutions for transient flux recordings in the apical region (100 μm from the root apex). The steady-state fluxes were continuously recorded for 10–20 min prior to the CdCl_2_ shock. CdCl_2_ stock (100 μM) was slowly added to the measuring solution until the final Cd^2+^ concentration reached 50 μM and transient kinetics of Cd^2+^ and Ca^2+^ were continuously for 20–30 min. Afterward, H_2_O_2_ (1.0 mM) was slowly added to the measuring solution and transient kinetics of Cd^2+^ and Ca^2+^ were restarted and continued for 20 min.

*Series 4: ROS scavenger*. NM and EM roots were pre-treated with or without 1, 3-Dimethyl-2-thiourea (DMTU, 5 mM, Chung et al., 2008; Sun et al., 2010) for 24 h in the presence and absence of 50 μM CdCl_2_. Then Cd^2+^ and Ca^2+^ fluxes were measured along root axes, 100–2,300 μm from the apex, at intervals of 200–300 μm.

*Series 5: External pH*. NM and EM roots were pre-treated with 50 μM CdCl_2_ for 24 h prior to flux measurements. Cd^2+^ and Ca^2+^ fluxes along root axes (100–2,300 μm from the apex) were recorded in Cd^2+^ or Ca^2+^ measuring solutions at pH 5.2, 6.2, or 7.2, respectively.

*Series 6: PM H^+^-ATPase inhibitor*. NM and EM roots were pre-treated with or without sodium orthovanadate (500 μM, Sun et al., 2010; Lu et al., 2013) for 24 h in the presence and absence of 50 μM CdCl_2_. Then H^+^, Cd^2+^, and Ca^2+^ fluxes were measured along root axes, 100–2,300 μm from the apex, at intervals of 200–300 μm. *P. involutus* isolates, MAJ and NAU, were exposed to 0 or 500 μM sodium orthovanadate for 24 h prior to a 30-min of continuous recording of H^+^ flux.

### Measurements of Net H_2_O_2_ Fluxes

An H_2_O_2_-sensititive microelectrode [tip diameter 2–3 μm, XY-DJ-502, Xuyue (Beijing) Science and Technology Co. Ltd., Beijing, China] was used to monitor H_2_O_2_ fluxes in EM and NM roots. H_2_O_2_ microelectrodes were prepared according to the method described by Twig et al. (2001). Before the measurement, H_2_O_2_ microelectrode was polarized at +0.60 V against an Ag/AgCl reference electrode. Thereafter, the microelectrodes were calibrated by the standard solution: 0.01, 0.1 and 1 mM H_2_O_2_. Roots sampled from control and CdCl_2_ (50 μM,30 min)-treated EM and NM plants were immobilized in the measuring solution (0.1 mM NaCl, 0.1 mM MgCl_2_, 0.1 mM CaCl_2_ and 0.5 mM KCl, pH was adjusted to 5.2 with KOH and HCl) and equilibrated for 25 min. The fluxes were recorded 100 μm from the apex and conducted along the root axis until 2300 μm, at intervals of 200–300 μm, and then calculated.

### Data Analysis

Ionic fluxes were calculated using the program JCal V3.2.1, a free MS Excel spreadsheet, which was developed by the Yue Xu^1^. The experimental data were subjected to SPSS (SPSS Statistics 17.0, 2008) for statistical tests and analyses. Unless otherwise stated, *P* < 0.05 was considered as significant.

## Results

### Cd^2+^-Altered Ion Flux Profiles in *Paxillus involutus*, and Roots of NM and EM Poplar

#### Cd^2+^ Fluxes

**FIGURE 1:**
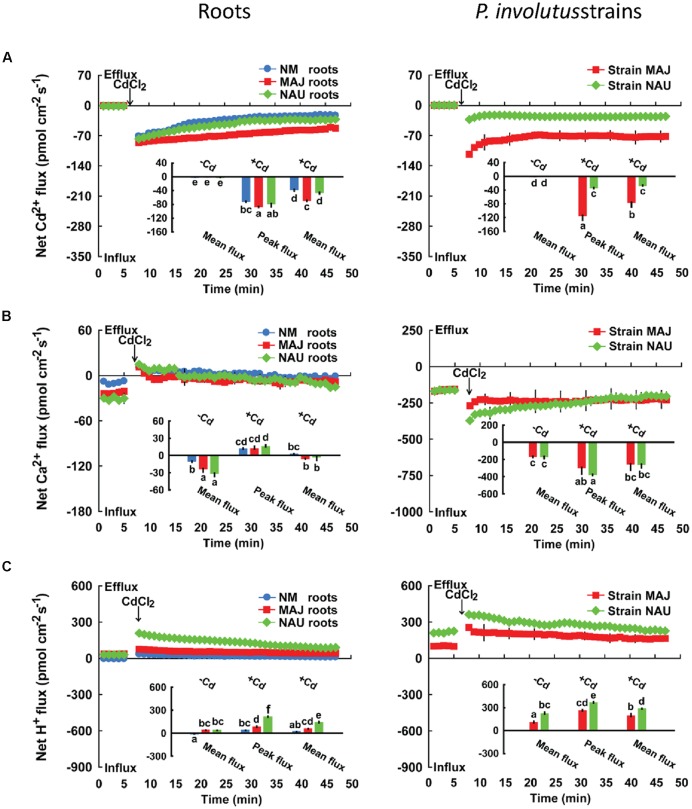
**Effects of CdCl_2_ on transient kinetics of Cd^2+^, Ca^2+^, and H^+^ in *Populus* × *canescens* roots and *Paxillus involutus* strains MAJ and NAU.** Cd^2+^ **(A)**, Ca^2+^ **(B)**, and H^+^ **(C)** kinetics were recorded before and after the required amount of 50 μM CdCl_2_ was introduced into the measuring chamber. Prior to the CdCl_2_ shock, steady-state fluxes of Cd^2+^, Ca^2+^, and H^+^ in ectomycorrhizal (MAJ and NAU) and non-mycorrhizal (NM) *P.* × *canescens* roots (measuring site was ca. 100 μm from the root tip) and *P. involutus* isolates were monitored for approximately 5 min. Transient kinetics of Cd^2+^, Ca^2+^, and H^+^ were recorded after the required amount of 50 μM CdCl_2_ was introduced into the measuring solution. Inserted sections show the peaking and/or mean values of Cd^2+^, Ca^2+^, and H^+^ flux before (-Cd) and after (+Cd) the addition of CdCl_2_. Columns represent the mean of four to five individual plants or axenic EM cultures (pelleted hyphae), and bars represent the standard error of the mean. Different letters, a, b, c, d, e, and f, indicate significant difference at *P* < 0.05 between treatments.

We recorded transient Cd^2+^ kinetics upon Cd^2+^ shock at the root apex (100 μm from the root tip; **Figure 1A**), where a vigorous ion flux (e.g., Na^+^, K^+^, Ca^2+^, Cd^2+^, Cl^-^) is usually observed in woody and herbaceous plants (Sun et al., 2009a,b; Li J. et al., 2012; Lu et al., 2013; Han et al., 2014). The addition of CdCl_2_ (50 μM) caused an immediate Cd^2+^ influx in both EM and NM roots which declined with increasing duration of Cd^2+^ exposure (40 min; **Figure 1A**). The peak and mean flux rate of Cd^2+^ in EM roots with MAJ were significantly (13.5 and 38.8%) higher than in NM roots or NAU-colonized roots (**Figure 1A**). Similar to the Cd^2+^ kinetics in EM roots, an instantaneous increase in the Cd^2+^ influx was detected in pure *P. involutus* mycelia after CdCl_2_ exposure (50 μM; **Figure 1A**). However, the fungal Cd^2+^ influx remained constant over the recording period (40 min; **Figure 1A**) with significantly higher flux rates in MAJ (75.4 pmol cm^-2^ s^-1^) than in NAU (25.9 pmol cm^-2^ s^-1^).

**FIGURE 2:**
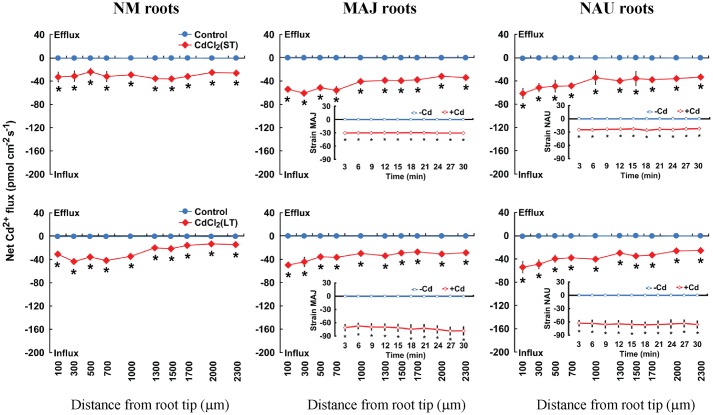
**Effects of CdCl_2_ on steady Cd^2+^ fluxes in *Populus* × *canescens* roots and *Paxillus involutus* strains MAJ and NAU.** *P. involutus* isolates, ectomycorrhizal (MAJ and NAU) and non-mycorrhizal (NM) *P.* × *canescens* plants were subjected to short-term (ST, 24 h) and long-term (LT, 7 d) exposure to 50 μM CdCl_2_, respectively. Control roots and axenic mycelia were well fertilized but treated without CdCl_2_. Cd^2+^ fluxes in poplar roots were measured along root axis, 100–2,300 μm from the apex, at intervals of 200–300 μm. Cd^2+^ fluxes of *P. involutus* isolates MAJ and NAU were measured along the surface of pelleted hyphae over a recording period of 30 min. Inserted sections show the Cd^2+^ fluxes in *P. involutus* isolates after short-term (ST, 24 h) or long-term (LT, 7 days) CdCl_2_ treatment. Each point is the mean of 4–5 individual plants or axenic EM cultures (pelleted hyphae), and bars represent the standard error of the mean. Asterisks denote significant difference at *P* < 0.05 between treatments.

After ST (24 h) or LT (7 days) exposure to 50 μM CdCl_2_ in hydroponic conditions, steady-state Cd^2+^ flux was recorded along root axis (100–2,300 μm from the apex) at intervals of 200–300 μm (**Figure 2**). In NM roots, ST and LT stress caused a net Cd^2+^ influx with an overall mean of 28.9 pmol cm^-2^ s^-1^ along the whole measured distance; LT treatment resulted in a higher flux rate at the region 100–1,000 μm from the apex than at more distant root positions (**Figure 2**). A similar trend was observed in the Cd^2+^-stressed EM roots, though mean Cd^2+^ fluxes in MAJ- and NAU-ectomycorrhizal roots were 43.1 and 32.0% higher than those of the NM roots under ST and LT stress (**Figure 2**). The mycelia of the two *P. involutus* strains, MAJ and NAU, exhibited a stable Cd^2+^ influx under ST and LT stress, although the CdCl_2_-induced Cd^2+^ influx was typically higher under LT conditions, 68.9 pmol cm^-2^ s^-1^, compared with ST treatment, 27.1 pmol cm^-2^ s^-1^ (**Figure 2**). Cd^2+^-induced alterations of Cd^2+^ flux were also examined in non-inoculated and *P. involutus*-inoculated roots after 7 days of co-culture. NAU- and MAJ-colonized roots showed larger flux rates than non-inoculated roots after ST Cd^2+^ stress (Supplementary Figure S1A).

Our data show that *P. involutus* mycelia and EM roots both exhibited an enhanced Cd^2+^ uptake upon Cd^2+^ shock, ST, or LT treatment (**Figures 1A** and **2**). Unexpectedly, the Cd^2+^ influx in EM roots did not show a high correlation to the flux rate of Cd^2+^ in fungal hyphae under various treatments (shock, ST, or LT, Supplementary Figure S2). However, a relatively high correlation between EM and NM roots was observed especially in response to Cd^2+^ shock (Supplementary Figure S2). This result supports that in the ectomycorrhizal symbioses the continuous Cd^2+^ entry detected by NMT microelectrodes depends on the uptake capacity of inner root cells and that in the plant–fungal interaction divergent regulation of fungal Cd^2+^ transport compared with pure mycelium must take place.

#### Ca^2+^ Fluxes

**FIGURE 3:**
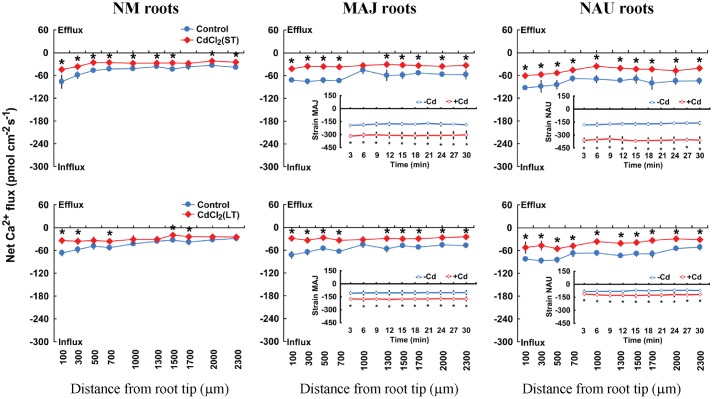
**Effects of CdCl_2_ on steady Ca^2+^ fluxes in *Populus* × *canescens* roots and *Paxillus involutus* strains MAJ and NAU.** *P. involutus* isolates, ectomycorrhizal (MAJ and NAU) and non-mycorrhizal (NM) *P.* × *canescens* plants were subjected to short-term (ST, 24 h) and long-term (LT, 7 days) exposure to 50 μM CdCl_2_, respectively. Control roots and axenic mycelia were well fertilized but treated without CdCl_2_. Ca^2+^ fluxes in poplar roots were measured along root axis, 100–2,300 μm from the apex, at intervals of 200–300 μm. Ca^2+^ fluxes of *P. involutus* isolates MAJ and NAU were measured along the surface of pelleted hyphae over a recording period of 30 min. Inserted sections show the Ca^2+^ fluxes in *P. involutus* isolates after short-term (ST, 24 h) or long-term (LT, 7 days) CdCl_2_ treatment. Each point is the mean of 4–5 individual plants or axenic EM cultures (pelleted hyphae), and bars represent the standard error of the mean. Asterisks denote significant difference at *P* < 0.05 between treatments.

In the absence of Cd^2+^ stress, poplar roots exhibited a net Ca^2+^ influx, with a greater flux rate in MAJ- and NAU-ectomycorrhizal roots, 26.9 pmol cm^-2^ s^-1^, than in NM roots, 9.6 pmol cm^-2^ s^-1^ (**Figure 1B**). Similarly, the mycelia of the two strains exhibited a stable and steady influx of Ca^2+^ (162.3 pmol cm^-2^ s^-1^), which is ca. 6.0-fold higher than that detected in EM roots (**Figure 1B**). CdCl_2_ shock (50 μM) caused a transient Ca^2+^ efflux in NM and EM roots with maximum values ranging from 10.9 to 14.8 pmol cm^-2^ s^-1^ (**Figure 1B**). Thereafter, the direction shifted toward an influx and the mean flux over the recording period then declined in EM roots, or displayed a net efflux in NM roots (**Figure 1B**). In contrast to NM and EM roots, Cd^2+^ addition markedly increased the Ca^2+^ influx in the hyphae of pure mycelium, typically with higher flux rates in strain NAU than in MAJ in the first 20 min of Cd^2+^ application (**Figure 1B**). Under ST and LT treatment, Cd^2+^ stress caused a marked decline of Ca^2+^ influx along the root axis (**Figure 3**). MAJ- and NAU-ectomycorrhizal roots maintained 40.5 and 20.6% higher Ca^2+^ fluxes than NM roots under ST and LT stress (**Figure 3**). In the hyphae of the two fungal strains, the Ca^2+^ influx was enhanced by ST and LT treatments (**Figure 3**), similar to the shock treatment (**Figure 1B**). We observed that the flux rate in the two strains declined with increasing duration of hydroponic culture regardless of control and Cd^2+^ treatments (**Figure 3**). Non-inoculated *P.* × *canescens* roots exhibited a net Ca^2+^ influx under unstressed control conditions and the Ca^2+^ influx was stimulated by 7 days inoculation with MAJ and NAU (Supplementary Figure S1B). ST-treated *P. involutus*-inoculated roots retained higher Ca^2+^ influx than non-inoculated roots although the Ca^2+^ influx in poplar roots was lowered by Cd^2+^ stress (Supplementary Figure S1B).

It has been suggested that the Ca^2+^ enrichment in EM roots was associated with the *P. involutus* fungal hyphae exhibiting a high capacity for Ca^2+^ uptake (**Figures 1B** and **3**; Li J. et al., 2012; Ma X. et al., 2014). However, the Ca^2+^ influx in EM roots was not evidently correlated to the flux rate of Ca^2+^ in fungal hyphae under Cd^2+^ shock, ST, or LT (Supplementary Figure S3). Unexpectedly, the Ca^2+^ flux in EM roots was even negatively correlated to the flux rate of Ca^2+^ in fungal hyphae after a shock treatment (Supplementary Figure S3). The observed correlation of Ca^2+^ fluxes between EM roots and NM roots (Supplementary Figure S3) supports that the Ca^2+^ flow was mainly the consequence of host roots in the Cd^2+^-stressed ectomycorrhizal symbioses.

#### Correlations between Cd^2+^ and Ca^2+^ Fluxes

**FIGURE 4:**
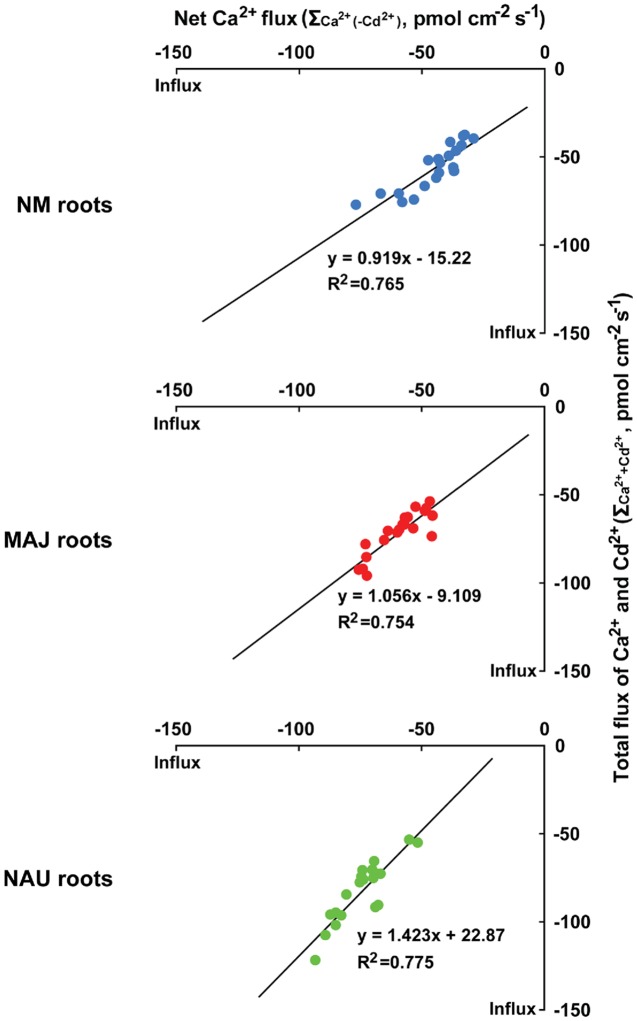
**The correlation between total fluxes of Ca^2+^ and Cd^2+^ (Σ_Ca_^2+^_+Cd_^2+^) in the presence of CdCl_2_ (50 μM, +Cd) and Ca^2+^ flux [Σ_Ca_^2+^_(-Cd_^2+^_)_] in the absence of CdCl_2_ (-Cd) in roots of ectomycorrhizal (MAJ and NAU) and non-mycorrhizal (NM) *Populus* × *canescens*.** Ectomycorrhizal (MAJ and NAU) and NM *P.* × *canescens* plants were subjected to short-term (ST, 24 h) and long-term (LT, 7 days) exposure to 50 μM CdCl_2_, respectively. Control roots were well fertilized but treated without CdCl_2_. Ca^2+^ and Cd^2+^ fluxes were measured along root axis, 100–2,300 μm from the apex, at intervals of 200–300 μm. Each point is the mean of 4–5 individual plants.

We analyzed the correlation between Cd^2+^ and Ca^2+^ fluxes as NM and EM roots took up these elements with a similar flux rate (**Figures 1A,B**, **2** and **3**). Under ST and LT stress conditions, the total flux rates of Cd^2+^ and Ca^2+^ in the presence of Cd^2+^ (=Σ_Ca_^2+^_+Cd_^2+^ with a molar ratio of Cd^2+^ to Ca^2+^ of 1:1) were 37.8–77.4 (NM), 54.1–96.2 (MAJ), and 53.7–122.1 pmol cm^-2^ s^-1^ (NAU), as calculated on the basis of **Figures 2** and **3** (Supplementary Figure S4). The relationships between Σ_Ca_^2+^_+Cd_^2+^ and Ca^2+^ flux in the absence of CdCl_2_ [Σ_Ca_^2+^_(-Cd_^2+^_)_] were highly significant and close to 1 for NM and MAJ colonized roots and slightly increased to 1.4 for NAU colonized roots (**Figure 4**). These suggest that the entry of Cd^2+^ and Ca^2+^ is mainly through the same pathway in NM and EM roots, mostly likely through Ca^2+^-permeable channels in the PM (see below).

#### H^+^ Fluxes

In the absence of CdCl_2_, EM roots showed a typical H^+^ efflux at the apex, which was 7.6-fold higher than that in NM roots (**Figure 1C**). CdCl_2_ (50 μM) shock stimulated H^+^ efflux in both NM and EM plants with a stronger response in EM than in NM roots (**Figure 1C**). Pure MAJ and NAU mycelia exhibited a net H^+^ efflux under control conditions similar to that observed for MAJ- and NAU-colonizing roots (**Figure 1C**). However, in pure mycelia the fluxes were 4.8-fold higher than in EM roots (**Figure 1C**). After exposure to CdCl_2_ (50 μM), hyphae exhibited a transient increase in the H^+^ efflux, which then remained constant during the period of recording (40 min; **Figure 1C**). Compared with strain MAJ, strain NAU exhibited higher H^+^ efflux irrespective of control or CdCl_2_ shock treatments (**Figure 1C**).

**FIGURE 5:**
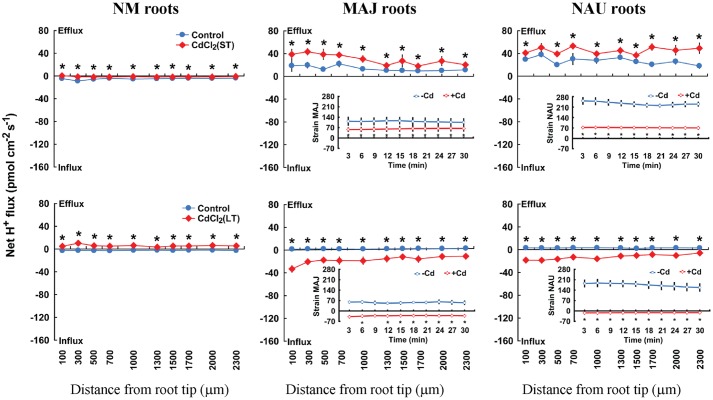
**Effects of CdCl_2_ on steady H^+^ fluxes in *Populus* × *canescens* roots and *Paxillus involutus* strains MAJ and NAU.** *P. involutus* isolates, ectomycorrhizal (MAJ and NAU) and non-mycorrhizal (NM) *P.* × *canescens* plants were subjected to short-term (ST, 24 h) and long-term (LT, 7 days) exposure to 50 μM CdCl_2_, respectively. Control roots and axenic mycelia were well fertilized but treated without CdCl_2_. H^+^ fluxes in poplar roots were measured along root axis, 100–2,300 μm from the apex, at intervals of 200–300 μm. H^+^ fluxes of *P. involutus* isolates MAJ and NAU were measured along the surface of pelleted hyphae over a recording period of 30 min. Inserted sections show the H^+^ fluxes in *P. involutus* isolates after short-term (ST, 24 h) or long-term (LT, 7 days) CdCl_2_ treatment. Each point is the mean of 4–5 individual plants or axenic EM cultures (pelleted hyphae), and bars represent the standard error of the mean. Asterisks denote significant difference at *P* < 0.05 between treatments.

Steady-state recordings on EM roots showed that the pattern of H^+^ flux in ST-stressed roots (50 μM CdCl_2_, 24 h) differed from those subjected to LT Cd^2+^ exposure (50 μM CdCl_2_, 7 days). Under ST conditions, CdCl_2_ (50 μM) stimulated H^+^ efflux in EM plants, whereas under LT conditions, EM roots showed a pronounced H^+^ influx (**Figure 5**). In NM roots, CdCl_2_ (50 μM) decreased H^+^ influx upon ST exposure or shifted it to a net H^+^ efflux under LT stress conditions (**Figure 5**). The pattern of H^+^ flux in the fungal mycelia differed from that in EM roots under ST stress (**Figure 5**). ST treatment reduced the efflux of H^+^ from the two fungal strains, which is contrast to EM roots where an enhanced H^+^ efflux was observed (**Figure 5**). LT stress caused a pronounced shift of H^+^ efflux to influx into pure mycelia of the two strains, similar to the finding in LT-stressed EM roots (**Figure 5**).

### Cd^2+^-Altered Flux Profiles of H_2_O_2_ in EM Roots

**FIGURE 6:**
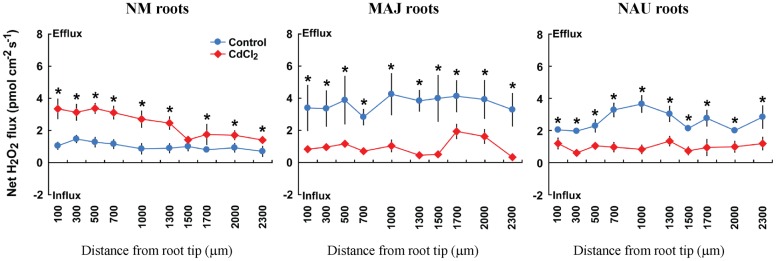
**Effects of CdCl_2_ on steady H_2_O_2_ flux in roots of ectomycorrhizal (MAJ and NAU) and non-mycorrhizal (NM) *Populus* × *canescens* plants.** Ectomycorrhizal (MAJ and NAU) and NM *P.* × *canescens* plants were subjected to 50 μM CdCl_2_ for 30 min. Control roots were well fertilized but treated without CdCl_2_. H_2_O_2_ flux was measured along root axis, 100–2,300 μm from the apex, at intervals of 200–300 μm. Each point is the mean of 4–5 individual plants, and bars represent the standard error of the mean. Asterisks denote significant difference at *P* < 0.05 between treatments.

H_2_O_2_-sensitive microprobes were used to detect the H_2_O_2_ response to Cd^2+^ exposure in NM and EM roots. In the absence of Cd^2+^, NM roots exhibited a stable H_2_O_2_ efflux (0.7–1.5 pmol cm^-2^ s^-1^) along the root axis; the mean flux rate increased 2.4-fold in response to Cd^2+^ treatment (50 μM CdCl_2_, 30 min, **Figure 6**). Ectomycorrhization of poplar roots with *P. involutus* stains, MAJ and NAU, resulted in a significant increase of H_2_O_2_ efflux along the roots (**Figure 6**). However, upon CdCl_2_ exposure EM roots displayed decreased H_2_O_2_ efflux in contrast to NM roots (**Figure 6**).

### Effects of Ca^2+^, H_2_O_2_, pH, and PM Transporter and Channel Inhibitors on Cd^2+^-Altered Ion Flux Profiles in EM Roots

#### Ca^2+^ and Ca^2+^ Channel Inhibitors

**FIGURE 7:**
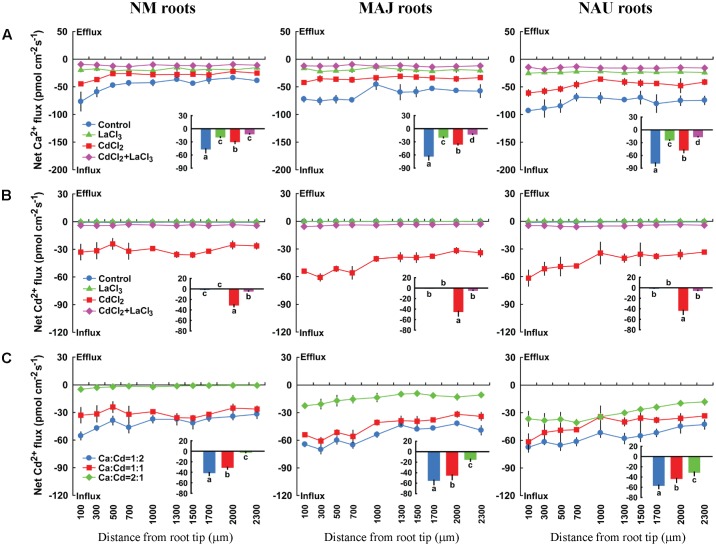
**Effects of LaCl_3_ and external Ca^2+^ on steady Cd^2+^ and/or Ca^2+^ fluxes in roots of ectomycorrhizal (MAJ and NAU) and non-mycorrhizal (NM) *Populus* × *canescens* plants under Cd^2+^ stress.** **(A,B)** Ectomycorrhizal (MAJ and NAU) and NM *P.* × *canescens* plants were subjected to 50 μM CdCl_2_ for 24 h in the presence and absence of 5 mM LaCl_3_. Control roots were well fertilized but treated without CdCl_2_ or LaCl_3_. **(C)** Ectomycorrhizal (MAJ and NAU) and NM *P.* × *canescens* plants were subjected to 50 μM CdCl_2_ for 24 h prior to Cd^2+^ flux recordings in the presence of CaCl_2_ (25 μM, 50 μM, or 100 μM; the ratio of Ca^2+^:Cd^2+^ was 1:2; 1:1, and 2:1). Ca^2+^ **(A)** and Cd^2+^ **(B,C)** fluxes were measured along root axes, 100–2,300 μm from the apex, at intervals of 200–300 μm. Each point is the mean of 4–5 individual plants and bars represent the standard error of the mean. Inserted sections show the mean flux rates and different letters, a, b, c, and d, indicate significant difference at *P* < 0.05 between treatments.

Here, pharmacological experiments were carried out to test whether putative Ca^2+^ channels inhibitors could inhibit Cd^2+^ influx in poplar roots. Four typical Ca^2+^ channels inhibitors, LaCl_3_, GdCl_3_, verapamil, and TEA effectively inhibited Ca^2+^ influx in NM and EM roots, regardless of Cd^2+^ treatments (**Figure 7A**, Supplementary Figures S5A, S6A, and S7A). LaCl_3_ restricted Cd^2+^ influx in CdCl_2_-treated NM and EM roots (**Figure 7B**). This suggests that Cd^2+^ is taken up through Ca^2+^-permeable channels because La^3+^ is able to block various types of Ca^2+^-permeable channels, including depolarisation-, hyperpolarisation-, elicitor-activated, and voltage-insensitive channels (Weiss, 1974; Pickard and Ding, 1993; Gelli and Blumwald, 1997; Piñeros and Tester, 1997; Zimmermann et al., 1997; White, 1998, 2000). Moreover, the other three Ca^2+^-permeable channel inhibitors, GdCl_3_, verapamil, and TEA, diminished Cd^2+^ influx to a similar extent as LaCl_3_-treated plants (**Figure 7B**, Supplementary Figures S5B, S6B, and S7B). Similarly, in the pure *P. involutus* mycelia, LaCl_3_ also effectively restricted influx of Ca^2+^ and Cd^2+^ or induced net efflux (Supplementary Figure S8).

Additionally, a co-application of Cd^2+^ and Ca^2+^ suppressed the entry of Cd^2+^ in NM and EM roots, and the restriction increased with the increasing fraction of Ca^2+^ in the mixture (Ca^2+^: Cd^2+^ = 1:2, 1:1, 2:1; **Figure 7C**). The mean Cd^2+^ flux decreased by 95.7% (NM), 72.1% (MAJ), and 45.5% (NAU) at a ratio of Ca^2+^:Cd^2+^ = 2:1, compared to a those with a higher Cd^2+^ fraction, Ca^2+^:Cd^2+^ = 1:2 (**Figure 7C**). These results suggest that the divalent cations, Cd^2+^ and Ca^2+^, competitively permeated the plasma membrane through Ca^2+^ channels. The lower reduction in Cd^2+^ influx in EM than in NM roots in the presence of Ca^2+^ (**Figure 7C**) reflects the high flow of Cd^2+^ through the activated Ca^2+^ channels.

We observed that the presence of Ca^2+^ in the measuring solution marginally lowered the Cd^2+^ signals (14.7–26.0%) detected by the Cd^2+^ microelectrodes filled with Cd^2+^ liquid ion exchanger (LIX) (Supplementary Table S1). In the absence of Ca^2+^, the working voltage of microelectrodes and the detected Cd^2+^ signals in Cd^2+^-treated roots were unstable and fluctuated greatly during the period of recording (data not shown). This behavior is presumably caused by the plant response to nutrient deficiency in the root medium (Li J. et al., 2012). In our study, Cd^2+^ electrodes exhibited higher sensitivity at 0.05 mM Ca^2+^ in the absence and presence of 0.1 mM K^+^ and 0.1 mM Mg^2+^ (Supplementary Table S1). The presence of nutrients, K^+^, Ca^2+^, and Mg^2+^, did not affect the accuracy of our conclusions relating to Cd^2+^ fluxes in NM and EM roots.

#### H_2_O_2_ and ROS Scavenger

**FIGURE 8:**
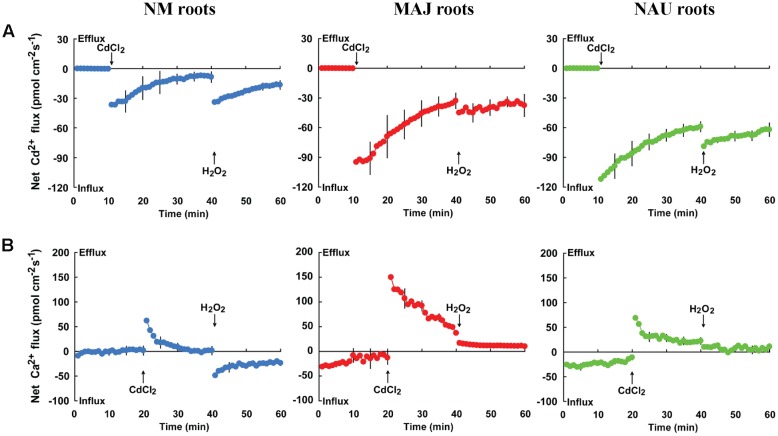
**Effects of CdCl_2_ and H_2_O_2_ on transient kinetics of Cd^2+^ and Ca^2+^ in roots of ectomycorrhizal (MAJ and NAU) and non-mycorrhizal (NM) *Populus* × *canescens*.** Cd^2+^ **(A)** and Ca^2+^ **(B)** kinetics were recorded before and after the required amount of 50 μM CdCl_2_ or 1.0 mM H_2_O_2_was introduced into the measuring chamber. Prior to the CdCl_2_ shock, steady-state fluxes of Cd^2+^ and Ca^2+^ were monitored at the apex (measuring site was ca. 100 μm from the root tip) for approximately 10–20 min. Transient kinetics of Cd^2+^ and Ca^2+^ were recorded after the required amount of 50 μM CdCl_2_ was introduced into the measuring solution. After 20–30 min continuous recording of Cd^2+^ and Ca^2+^ fluxes, Cd^2+^ and Ca^2+^ kinetics were recorded for 20 min after 1.0 mM H_2_O_2_ was introduced into the measuring solution. Each point represents the mean of 4–5 individual plants and bars represent the standard error of the mean.

To investigate whether Cd^2+^ entry through Ca^2+^-permeable channels is activated by H_2_O_2_, we examined the effects of hydrogen peroxide and the ROS (reactive oxygen species) scavenger DMTU on Cd^2+^ and Ca^2+^ fluxes. Transient kinetic recordings showed that Cd^2+^ shock caused an immediate increase of Cd^2+^ influx but enhanced Ca^2+^ efflux in NM and EM roots (**Figure 8**). The flux rates of Cd^2+^ and Ca^2+^ decreased with prolonged exposure time (**Figure 8**). Notably, Cd^2+^ influx markedly increased upon H_2_O_2_ shock (1.0 mM) in both NM and EM roots (**Figure 8A**). However, the Cd^2+^-elicited Ca^2+^ efflux was reduced by H_2_O_2_ in EM roots or shifted to a net influx in NM roots (**Figure 8B**). These results suggest that H_2_O_2_ stimulated the entry of Cd^2+^ and Ca^2+^, presumably through the plasma membrane Ca^2+^ channels of the roots.

**FIGURE 9:**
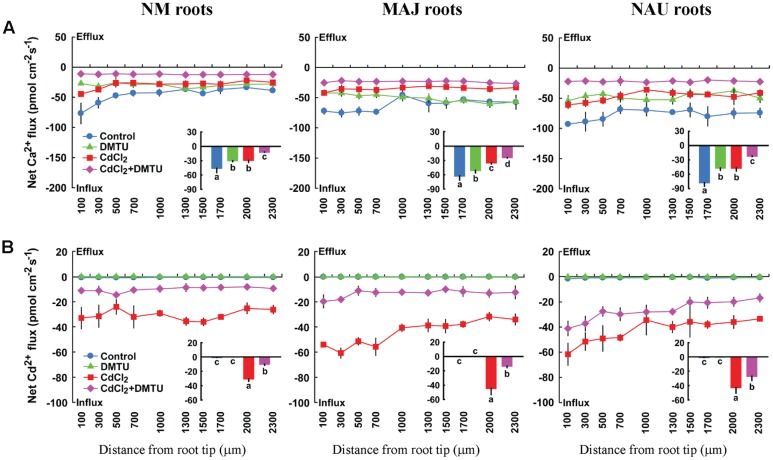
**Effects of DMTU on steady Ca^2+^ and Cd^2+^ fluxes in roots of ectomycorrhizal (MAJ and NAU) and non-mycorrhizal (NM) *Populus* × *canescens* under Cd^2+^ stress.** Ectomycorrhizal (MAJ and NAU) and NM *P.* × *canescens* plants were subjected to 0 or 50 μM CdCl_2_ for 24 h in the presence or absence of 5 mM DMTU. Ca^2+^ **(A)** and Cd^2+^ **(B)** fluxes were measured along root axes, 100–2,300 μm from the apex, at intervals of 200–300 μm. Each point is the mean of 4–5 individual plants and bars represent the standard error of the mean. Inserted sections show the mean flux rates and different letters, a, b, c, and d, indicate significant difference at *P* < 0.05 between treatments.

Ca^2+^ influx in NM and EM roots were suppressed by the ROS scavenger, DMTU (5 mM), irrespective of the presence and absence of Cd^2+^ (**Figure 9A**). Similarly, the supplement of DMTU significantly reduced the influx of Cd^2+^ in NM and EM roots (**Figure 9B**). These data indicated that H_2_O_2_ play a crucial role in accelerating the influx of Ca^2+^ and Cd^2+^, which is accordance to the results obtained by direct H_2_O_2_ applications (**Figure 8**).

#### External pH and H^+^-ATPase Inhibitor

**FIGURE 10:**
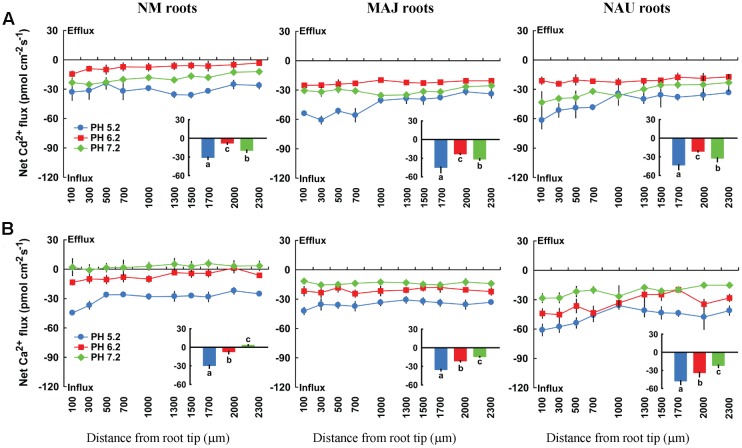
**Effects of pH on steady Cd^2+^ and Ca^2+^fluxes in roots of ectomycorrhizal (MAJ and NAU) and non-mycorrhizal (NM) *Populus* × *canescens* under Cd^2+^ stress.** Ectomycorrhizal (MAJ and NAU) and NM *P.* × *canescens* plants were subjected to 50 μM CdCl_2_ for 24 h prior to flux recordings at pH 5.2, 6.2 or 7.2. Cd^2+^ **(A)** and Ca^2+^ **(B)** fluxes were measured along root axes, 100–2,300 μm from the apex, at intervals of 200–300 μm. Each point is the mean of four to five individual plants and bars represent the standard error of the mean. Inserted sections show the mean flux rates and different letters, a, b, and c, indicate significant difference at *P* < 0.05 between treatments.

Fluxes of Cd^2+^ and Ca^2+^ depend on external pH. An acidic environment accelerated Cd^2+^ and Ca^2+^ influxes in both NM and EM roots with the strongest influx at pH 5.2 and the lowest at pH 6.2 or a neutral pH, 7.2 (**Figure 10**). Moreover, we noticed that the pH effects on fluxes of Cd^2+^ and Ca^2+^ were more pronounced in NM roots than in EM roots (**Figure 10**). Compared to an acidic environment (pH 5.2), the mean flux rate of the divalent cations decreased by 45.8% (Ca^2+^) and 38.8% (Cd^2+^) in EM roots under pH 6.2–7.2 (**Figure 10**). In NM roots, the increasing pH lowered Cd^2+^ influxes by 56.5% or even reversed the rectifications of Ca^2+^ (influx → efflux) at a neutral pH, 7.2 (**Figure 10**). The less reduced influx of Ca^2+^ and Cd^2+^ in EM roots at pH 6.2 or 7.2 was due to the high H^+^-pumping activity in the PM (see below).

**FIGURE 11:**
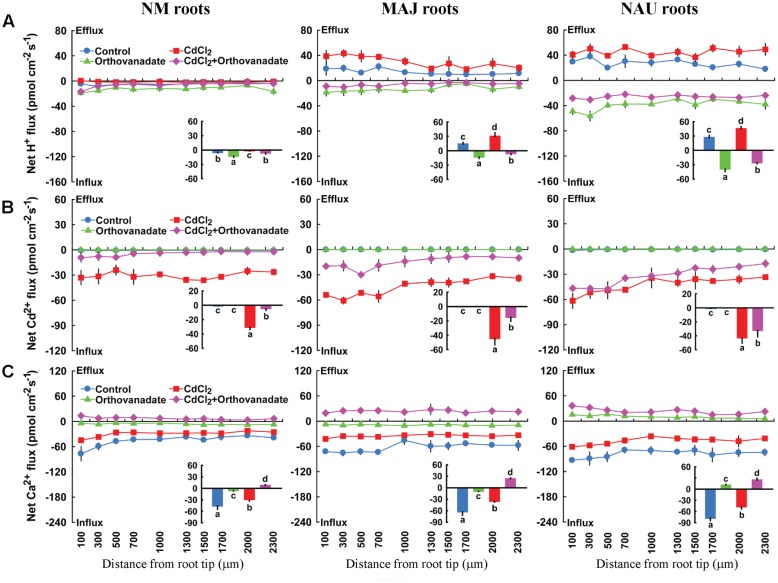
**Effects of sodium orthovanadate on steady H^+^, Cd^2+^, and Ca^2+^ fluxes in roots of ectomycorrhizal (MAJ and NAU) and non-mycorrhizal (NM) *Populus* × *canescens* under Cd^2+^ stress.** Ectomycorrhizal (MAJ and NAU) and NM *P.* × *canescens* plants were subjected to 0 or 50 μM CdCl_2_ for 24 h in the presence and absence of 500 μM sodium orthovanadate. H^+^ **(A)**, Cd^2+^ **(B)**, and Ca^2+^ **(C)** fluxes were measured along root axes, 100–2,300 μm from the apex, at intervals of 200–300 μm. Each point is the mean of 4–5 individual plants and bars represent the standard error of the mean. Inserted sections show the mean flux rates and different letters, a, b, c, and d, indicate significant difference at *P* < 0.05 between treatments.

Sodium orthovanadate (500 μM), the specific inhibitor of PM H^+^-ATPase, increased the H^+^ influx in NM roots slightly, but caused a drastic shift from H^+^ efflux toward influx in both EM roots and *P. involutus* mycelia, irrespective of Cd^2+^ treatment (**Figure 11A**, Supplementary Figure S9). Sodium orthovanadate significantly reduced the Cd^2+^ influx along the roots in Cd^2+^-treated NM and EM plants (**Figure 11B**). In the absence of Cd^2+^, the PM H^+^-ATPase inhibitor reduced Ca^2+^ influx in NM and MAJ-ectomycorrhizal roots or shifted to efflux in NAU-ectomycorrhizal roots (**Figure 11C**). The inhibition of Ca^2+^ influx by sodium orthovanadate was more pronounced in the presence of Cd^2+^: the H^+^-pump inhibitor reversed the rectifications of Ca^2+^ from influx to efflux in NM and EM roots (**Figure 11C**).

## Discussion

### Colonization of *P.* × *canescens* Roots with *Paxillus involutus* Stimulates Cd^2+^ Uptake under Cd^2+^ Stress

The woody Cd^2+^-hyperaccumulator *P.* × *canescens* exhibited a vigorous Cd^2+^ uptake after a 50 μM CdCl_2_ shock (40 min), ST (24 h), and LT (7 days) treatment (**Figures 1A** and **2**). The result is consistent with previous findings where *P.* × *canescens* roots exhibited a high Cd^2+^ uptake after 40 days of CdSO_4_ exposure (50 μM, Ma Y. et al., 2014). Similarly, a high entry of Cd^2+^ was recorded in hyperaccumulating ecotypes of *Sedum alfredii* (Lu et al., 2010; Sun et al., 2013a) and *Suaeda salsa* under Cd^2+^ stress (Li et al., 2012a). An important result was that EM roots exhibited higher Cd^2+^ influx than NM roots irrespective of Cd^2+^ stress conditions, shock, ST, and LT (**Figures 1A** and **2**). Substantial evidence indicates that Cd^2+^ can be enriched in ectomycorrhizal plants (Sell et al., 2005; Baum et al., 2006; Krpata et al., 2008, 2009; Sousa et al., 2012; Ma Y. et al., 2014). The enhanced Cd^2+^ uptake in EM roots is partly due to the capacity of the fungus to take up Cd^2+^ because CdCl_2_ shock resulted in a net Cd^2+^ influx in the mycelia of the two *P. involutus* strains and the flux rate increased with the prolonged duration of CdCl_2_ treatment from 24 h to 7 days (**Figures 1A** and **2**). In liquid cultures, *P. involutus* cultures also showed high capacities for Cd^2+^ accumulation (Ott et al., 2002). *P. involutus* could bind Cd^2+^ onto the cell walls or accumulate the metal in the vacuolar compartment (Blaudez et al., 2000; Ott et al., 2002). Moreover, the ectomycorrhizal fungus appears to detoxify high concentrations of Cd^2+^ by (i) the chelation of metal ions in the cytosol with thiol-containing compounds, e.g., glutathione, phytochelatins, or metallothioneins (Courbot et al., 2004; Jacob et al., 2004), and (ii) activation of antioxidative defense system (Jacob et al., 2001; Ott et al., 2002). Our pharmacological data revealed that Cd^2+^ entered the fungal hyphae mainly through PM Ca^2+^ channels because the influx was suppressed by LaCl_3_, a Ca^2+^ channel blocker (Supplementary Figure S8B). Therefore, Cd^2+^ enriched by ectomycorrhizal hyphae is thought to be transferred to the host roots, probably through the apoplastic space during the period of Cd^2+^ stress.

There were marked differences between the two strains in Cd^2+^ uptake given the shock treatment (**Figure 1A**). Pure fungal mycelium of MAJ accumulated Cd^2+^ with a higher rate than NAU (**Figure 1A**). In the *P. involutus*-ectomycorrhizal symbioses, the incompatible fungal isolate NAU is unable to induce a functional ectomycorrhizae while MAJ forms a typical Hartig net with the roots of *P.* × *canescens* (Gafur et al., 2004). Thus, in MAJ-colonized roots the host cells might have been more accessible to Cd^2+^. In accordance, MAJ roots exhibited a higher influx than NAU roots after the onset of CdCl_2_ shock (**Figure 1A**). However, Cd^2+^ influx into NAU-colonized roots was similar to that of MAJ-colonized roots during ST or LT Cd^2+^ treatment (**Figure 2**). This was likely due to (i) similar capacities for Cd^2+^ uptake of MAJ and NAU hyphae during a 24-h or 7-days of Cd^2+^ exposure (**Figure 2**), or (ii) similar uptake capacity of the fungus-ensheathed inner root cells (**Figure 2**). The observed correlation between EM and NM roots showed that the continuous Cd^2+^ flow was mainly the consequence of host roots in the Cd^2+^-stressed ectomycorrhizal symbioses during a prolonged period of Cd^2+^ exposure (24 h to 7 days; Supplementary Figure S2).

### *Paxillus involutus*-Ectomycorrhizas Enhance Cd^2+^ Influx through Ca^2+^-Permeable Channels in the Plasma Membrane

Our data revealed that the entry of Cd^2+^ is likely mediated through PM Ca^2+^ channels in the fungal hyphae and poplar roots, and *P. involutus*-ectomycorrhizas facilitated the channel-mediated Cd^2+^ influx under Cd^2+^ stress. The experimental evidence for these conclusions is briefly listed below.

(1) The addition of Cd^2+^ resulted in an immediate influx of Cd^2+^ in NM roots, and the flux was more pronounced in EM roots (**Figure 1A**). Rapid entry of Cd^2+^ is generally through PM ion channels that are permeable to Cd^2+^ (The first 1–2 min flux recordings were discarded to diminish the diffusion effect of stock addition in roots and fungal mycelia). Our pharmacological data revealed that the net Cd^2+^ influx in CdCl_2_-stressed NM and EM roots was strongly suppressed by typical Ca^2+^ channel blockers, such as LaCl_3_, GdCl_3_, verapamil, and TEA (**Figure 7B**, Supplementary Figures S5B, S6B, and S7B). Moreover, in *P. involutus* mycelium the CdCl_2_-elicited influx of Cd^2+^ was also inhibited by LaCl_3_ (Supplementary Figure S8B). These results suggest that under CdCl_2_ stress Cd^2+^ enters fungal and root tissues through PM Ca^2+^ channels.
(2) Cd^2+^ treatments (shock, ST, and LT) affected the uptake of Ca^2+^ in poplar roots (**Figures 1B** and **3**), while the influx of Cd^2+^ declined with increasing the concentration of Ca^2+^ when NM and EM roots were subjected to the concomitant application of Cd^2+^ and Ca^2+^ (**Figure 7C**). Similarly, the Cd^2+^ influx was affected by the presence of Ca^2+^ in two contrasting (hyperaccumulating and non-hyperaccumulating) *Sedum alfredii* ecotypes (Lu et al., 2010). It was suggested that Ca^2+^ and Cd^2+^ ions compete for the binding sites of transporters (Gussarsson et al., 1996; Rodríguez-Serrano et al., 2009). Our transient kinetics showed that Cd^2+^ exposure blocked the Ca^2+^ influx and caused an immediate change in the rectification of Ca^2+^ from influx to efflux (**Figures 1B** and **8B**). This suggests that Cd^2+^ ions competed with Ca^2+^ to penetrate across PM Ca^2+^ channels that are permeable to divalent cations (Perfus-Barbeoch et al., 2002).
(3) In ST- and LT-stressed NM and EM roots, the total flux rates of Cd^2+^ and Ca^2+^ in the presence of Cd^2+^ (Σ_Ca_^2+^_+Cd_^2+^) were nearly equal to the flux rate of Ca^2+^ in the absence of Cd^2+^ stress (Σ_Ca_^2+^_(-Cd_^2+^_)_; Supplementary Figure S4). Moreover, the correlations between Σ_Ca_^2+^_+Cd_^2+^ and Σ_Ca_^2+^_(-Cd_^2+^_)_ (**Figure 4**) suggest that Cd^2+^ ions enter NM and EM roots mainly through Ca^2+^-permeable channels in the PM.

Collectively, under CdCl_2_ stress Cd^2+^ ions could penetrate the PM Ca^2+^ channels in fungal hyphae and in *P.* × *canescens* roots. At present we cannot exclude the possibility that Cd^2+^ penetrated the PM through transporters for Cd^2+^ (Ma Y. et al., 2014; He et al., 2015) or other nutritional ions (Gussarsson et al., 1996; Cohen et al., 1998; Zhao et al., 2002; Cosio et al., 2004; Clemens, 2006), because (1) the four types of Ca^2+^ channel inhibitors applied here were not able to fully block the Cd^2+^ influx in NM and EM roots (**Figure 7B**, Supplementary Figures S5B, S6B and S7B), and (2) the total flux of Cd^2+^ and Ca^2+^ (Σ_Ca_^2+^_+Cd_^2+^, molar ratio of Cd^2+^ to Ca^2+^ is 1:1) under ST and LT Cd^2+^ stress was 10.9–27.7% higher than the flux rate of Ca^2+^ under non-Cd^2+^ conditions (**Figure 4**, Supplementary Figure S4). This implies that a small fraction of Cd^2+^ ions penetrated the PM through other channels and transporters.

Plasma membrane Ca^2+^ channels in *P. involutus* hyphae maybe more permeable to Cd^2+^ compared to the channels in *P.* × *canescens* roots as the fungal mycelium displayed a typical higher Ca^2+^ influx than poplar roots under control and Cd^2+^-stress conditions (**Figures 1B** and **3**). We cannot discriminate between the channels of the fungus and those of the plant in the ectomycorrhizal symbiosis, but the Cd^2+^ and Ca^2+^ fluxes in EM roots appear to mainly reflect the response of the host plants to Cd^2+^ stress because (1) EM roots exhibited a different pattern from the *P. involutus* mycelia in enhancing Ca^2+^ and Cd^2+^ uptake under hydroponic Cd^2+^ conditions. Cd^2+^-shocked MAJ and NAU fungal strains usually displayed a stable Cd^2+^ influx with the exception of an initial transient increase (**Figure 1A**). However, EM roots showed a declined Cd^2+^ influx over the duration of Cd^2+^ exposure, similar to the Cd^2+^ kinetics in NM roots (**Figure 1A**). Moreover, the Cd^2+^ influx in the mycelia of the two *P. involutus* strains increased with the prolonged CdCl_2_ exposure from 24 h to 7 days (from 23.9 to 72.7 pmol cm^-2^ s^-1^; **Figure 2**). In contrast, the Cd^2+^ fluxes in EM roots were relatively stable under ST (43.7 ± 8.4 pmol cm^-2^ s^-1^) and LT treatments (35.9 ± 6.0 pmol cm^-2^ s^-1^; **Figure 2**). ST, LT, and Cd^2+^ shock increased the Ca^2+^ influx in *P. involutus* mycelia, while the Ca^2+^ influx in EM roots was declined by these Cd^2+^ treatments (**Figures 1B** and **3**). (2) NMT data showed that ion fluxes in mature *P.* × *canescens–P. involutus* symbiotic associations bear a striking resemblance to the ST inoculated roots (Supplementary Figure S1). Similar findings have been previously reported in a salt stress study where *P.* × *canescens* roots were inoculated with *P. involutus* for 10 and 20 days (Ma X. et al., 2014). At early stages of fungal co-culture the Cd^2+^ and Ca^2+^ influx is mostly the result of host properties. Therefore, the Cd^2+^ and Ca^2+^ stimulation in *P. involutus*-ectomycorrhizal roots reflects the enhanced root uptake ability. (3) The correlation analyses revealed that Cd^2+^ and Ca^2+^ influxes in EM roots show a significant relationship with NM roots but not with fungal mycelia under various Cd^2+^ treatments (shock, ST, and LT; Supplementary Figures S2 and S3). Taken together, these data suggest that the continuous flow of Cd^2+^ and Ca^2+^ in EM roots detected by NMT microelectrodes was largely driven by the host and that the fungal partner enhanced fluxes leading to enriched Cd^2+^ and Ca^2+^ concentrations.

The observed patterns of Cd^2+^ and Ca^2+^ fluxes upon Cd^2+^ exposure could be explained by channel-mediated ion fluxes. NMT data show that the Ca^2+^ flux in EM roots was negatively correlated with the Ca^2+^ influx in fungal hyphae upon Cd^2+^ shock treatment (Supplementary Figure S3). This is presumably the result of Cd^2+^-Ca^2+^ competition across the Ca^2+^ channels in the root PM. After being exposed to Cd^2+^ shock, Ca^2+^ entry was enhanced in the hyphae (**Figure 1B**). However, the fungal hyphae which were enriched in Ca^2+^ ions, were unable to deliver Ca^2+^ to the root cells because the Cd^2+^ ions competitively inhibited the entry of Ca^2+^ through the PM channels. As a result, the high influx of Ca^2+^ through fungal hyphae led to an apparently greater Ca^2+^ efflux in Cd^2+^-exposed EM roots (**Figures 1B** and **8B**).

*Paxillus involutus* colonization enhanced the uptake of Cd^2+^ under shock, ST, and LT stress, compared to NM roots (**Figures 1A** and **2**). The increased entry of Cd^2+^ is likely due to the activation of PM Ca^2+^ channels in the ectomycorrhizas. The stimulated Ca^2+^ influx by *P. involutus* inoculation revealed the activation of PM Ca^2+^ channels since the ectomycorrhiza-enhanced entry of Ca^2+^ was suppressed by Ca^2+^ channel blockers (LaCl_3_, GdCl_3_, verapamil, or TEA; **Figure 7A**, Supplementary Figures S5A, S6A, and S7A). The activated PM Ca^2+^ channels allowed the entry of Cd^2+^ in addition to Ca^2+^ under Cd^2+^ stress (**Figures 1A** and **2**).

### Hydrogen Peroxide Induced by CdCl_2_ and Fungal Colonization Stimulates Cd^2+^ Influx through PM Ca^2+^ Channels

After being subjected to CdCl_2_ exposure, NM roots displayed an increased H_2_O_2_ efflux along the root axis (**Figure 6**). It is well documented that Cd^2+^ induced accumulation of H_2_O_2_ in pine roots (Schützendübel et al., 2001), *P* × *canescens* roots (Schützendübel et al., 2002), and in suspension cultures of tobacco (Piqueras et al., 1999) and *P. euphratica* (Sun et al., 2013b; Han et al., 2016). H_2_O_2_ efflux was evident in EM roots irrespective of the presence or absence of Cd^2+^ treatments (**Figure 6**). Our results suggest that the Cd^2+^ influx through PM Ca^2+^ channels is stimulated by H_2_O_2_ in NM and EM roots. The experimental evidence and explanations are briefly listed here.

(1) H_2_O_2_ (1.0 mM) exhibited an enhancement on Ca^2+^ influx in NM and EM roots (**Figure 8B**). Pei et al. (2000) showed that H_2_O_2_ (0.05–5.0 mM) activates Ca^2+^ currents through PM Ca^2+^ channels of *Arabidopsis thaliana* guard cells. Moreover, H_2_O_2_ increased Ca^2+^ influx across the PM in *P. euphratica* cells (Sun et al., 2010), roots of *A. thaliana* (Demidchik et al., 2007) and mangroves (Lu et al., 2013). Furthermore, the H_2_O_2_-stimulated entry of Ca^2+^ in *P. euphratica* cells was inhibited by LaCl_3_ (Sun et al., 2010). In this study, Cd^2+^ influx in NM and EM roots was significantly enhanced after exposure to 1.0 mM H_2_O_2_ (**Figure 8A**). This finding is in agreement with Sun et al. (2013b) and Han et al. (2016), who found that H_2_O_2_ (3.0 mM) stimulated entry of Cd^2+^ into *P. euphratica* cells. In addition, the Cd^2+^ influx was blocked by LaCl_3_ in CdCl_2_-stressed roots (**Figure 7B**). These results suggest that H_2_O_2_ stimulates the influx of Cd^2+^ and Ca^2+^ through Ca^2+^-permeable channels in the PM.
(2) The H_2_O_2_ induction of Cd^2+^ resembles the pattern of Ca^2+^ kinetics in response to H_2_O_2_ (**Figure 8**). Moreover, Cd^2+^ and Ca^2+^ influx in NM and EM roots were both suppressed by the ROS scavenger, DMTU (**Figure 9**). Similarly, Sun et al. (2013b) showed that the entry of Cd^2+^ into *P. euphratica* cells was reduced when a H_2_O_2_ scavenger, catalase, was applied.

Taken together, these results suggest that Cd^2+^ and Ca^2+^ ions enter NM and EM roots by the same pathway involving PM Ca^2+^ channels that are activated by Cd^2+^-elicited H_2_O_2_.

The high Cd^2+^ influx in EM roots resulted from the pronounced activation of PM Ca^2+^ channels that were stimulated, at least in part, by the fungal-elicited H_2_O_2_. Compared to NM roots, MAJ- and NAU-ectomycorrhizal roots displayed a significant higher H_2_O_2_ efflux in the absence of Cd^2+^ stress (**Figure 6**), suggesting that the inoculation with *P. involutus* caused a strong production of H_2_O_2_ in EM roots. This finding agrees with Gafur et al. (2004) and Langenfeld-Heyser et al. (2007), who detected strong H_2_O_2_ accumulation in the outer hyphae mantle of compatible (MAJ) and incompatible (NAU) interactions. H_2_O_2_ production in the hyphae is suggested to regulate host’s root growth, defense against other invading microbes, and increasing plant-innate immunity (Salzer et al., 1999; Gafur et al., 2004). In our study, H_2_O_2_ produced in the ectomycorrhizae accelerated the influx of Ca^2+^ in the absence of Cd^2+^, whereas it increased entry of Cd^2+^ in the presence of high external Cd^2+^ (**Figure 8**). ROS scavenging by DMTU simultaneously decreased Ca^2+^ and Cd^2+^ influxes along the root axis of EM plants (**Figure 9**). These observations suggest that H_2_O_2_ produced in compatible (MAJ) and incompetent (NAU) ectomycorrhizal associations activated Ca^2+^ permeable channels, which allowed the entry of Cd^2+^ under Cd^2+^ stress.

We noticed that the H_2_O_2_ efflux in MAJ and NAU-ectomycorrhizal roots was lowered by Cd^2+^ stress (**Figure 6**). This reduction may have resulted from the activation of antioxidant enzymes and increased amounts of ROS scavengers produced as a defense response. It has been repeatedly shown that the antioxidant enzyme activities are activated under heavy metal stresses (Schützendübel et al., 2001, 2002; Rozpądek et al., 2014; Chen et al., 2015; Tan et al., 2015). The enhanced activities of superoxide dismutase, peroxidase, catalase, and ascorbate peroxidase play an important role in scavenging the Cd^2+^-elicited H_2_O_2_ in plants (Garg and Aggarwal, 2012; Anjum et al., 2015; Tan et al., 2015). To combat Cd^2+^-induced superoxide and H_2_O_2_, *P.* × *canescens* plants were found to rely mainly on antioxidant enzymes and the formation of the potential radical scavenging molecules, such as proline, sugar alcohols and soluble phenolics (He et al., 2011). However, the lowered H_2_O_2_ efflux in EM roots (**Figure 6**) did not reduce the Cd^2+^-elicited entry of Cd^2+^, because (i) the fungal-elicited H_2_O_2_ had already activated the Ca^2+^-channels before the Cd^2+^ addition, and/or (ii) the H_2_O_2_ level is still high enough to activate the channels under Cd^2+^ stress. The observation that stressed EM roots still contain high concentrations of H_2_O_2_ in the hyphae (Langenfeld-Heyser et al., 2007) supports these speculations.

### PM H^+^-ATPase Activated by Cd^2+^ and Fungal Colonization Stimulates Cd^2+^ Influx through PM Ca^2+^ Channels

In addition to H_2_O_2_, PM H^+^-ATPase activated by Cd^2+^ and enhanced by fungal colonization also accelerated Cd^2+^ influx through PM Ca^2+^ channels in NM and EM roots. PM H^+^-ATPases pump protons into the external medium to maintain an electrochemical H^+^ gradient across the PM (Blumwald et al., 2000; Zhu, 2003). Krämer (2010) suggested that H^+^-ATPases play an important role in adaptation of plants to heavy metal stress. The finding that the net H^+^ efflux in fungal mycelia and EM roots was markedly reduced by a specific inhibitor of PM H^+^-ATPase (sodium orthovanadate) in the presence and absence of Cd^2+^ stress (**Figure 11A**, Supplementary Figure S9) supports that the vigorous H^+^ efflux is the consequence of H^+^-ATPase activity. Accordingly, the increased H^+^ efflux upon Cd^2+^ shock (NM, MAJ and NAU roots; **Figure 1C**), ST (MAJ and NAU roots; **Figure 5**) and LT stress (NM roots; **Figure 5**) indicates the activated H^+^-pumping activity. In NM and EM roots, Cd^2+^ exposure led to a marked upregulation of *HA2.1* and *AHA10.1*, two important genes encoding PM H^+^-ATPases (Ma Y. et al., 2014). The activation of PM H^+^-ATPase by Cd^2+^ is likely associated with the Cd^2+^-elicited H_2_O_2_, since (i) H_2_O_2_ increased H^+^ pumping activity in *P. euphratica* callus cells (Sun et al., 2010), in roots of *P. euphratica* (Sun et al., 2010) and secretor and non-secretor mangrove species (Lu et al., 2013; Lang et al., 2014), and (ii) the expression of genes encoding PM H^+^-ATPase are stimulated by H_2_O_2_ in *Cucumis sativus* roots (Janicka-Russak and Kabala, 2012; Janicka-Russak et al., 2012).

The activated PM H^+^-ATPase enabled NM and EM roots to maintain an acidic environment, which favors the entry of Cd^2+^ across the PM (**Figure 10A**). Similarly, He et al. (2015) showed that pH 5.5 accelerates Cd^2+^ influx into poplar roots compared to pH 4.0 or pH 7.0. Moreover, the Cd^2+^ influx was markedly suppressed by the application of sodium vanadate, an inhibitor of PM H^+^-ATPase (**Figure 11B**). These results indicate that the PM H^+^-pumps play a crucial role in enhancing the entry of Cd^2+^ (Ma Y. et al., 2014). Accordingly, NMT profiles of NM and EM roots showed that the maximum influx of Ca^2+^ was observed at pH 5.2 (**Figure 10B**), and that Ca^2+^ influx was blocked by sodium vanadate (**Figure 11C**). Therefore, we infer that Cd^2+^ activated H^+^-pumping in the PM, which led to hyperpolarization of the PM and increased Cd^2+^ influx through hyperpolarization-activated Ca^2+^ channels (HACCs). However, at present we cannot exclude the possibility that Cd^2+^ ions also penetrated through depolarization-activated (DACCs) and voltage-independent Ca^2+^ channels (VICCs), because the inhibitor of PM H^+^-ATPase, sodium vanadate, could not fully block the Cd^2+^ influx in NM and EM roots (**Figure 11B**). It has been shown that NSCCs co-exist with HACCs in the root cell plasma membrane to mediate the entry of Ca^2+^, but the two Ca^2+^ influx routes differ in their voltage sensitivity (Demidchik et al., 2002).

Ectomycorrhizal *Populus* × *canescens* show highly activated H^+^-pumping activity in the PM, which favors the Cd^2+^ influx through HACCs. Our NMT data showed that colonization of *P.* × *canescens* with *P. involutus* caused a marked H^+^ efflux (**Figures 1C**, **5**, and **11A**), suggesting that the fungal colonization could activate the PM H^+^-ATPase in ectomycorrhizas. This is consistent to our previous studies (Li J. et al., 2012; Ma X. et al., 2014). It has been documented that some host PM H^+^-ATPase isoforms show high activity in arbuscular mycorrhizal associations (Ramos et al., 2005; Rosewarne et al., 2007). Obviously, H^+^-pumping activity was activated by Cd^2+^ shock and ST exposure, as the H^+^ efflux in MAJ- and NAU-ectomycorrhizal roots were significantly higher than the NM roots (**Figures 1C** and **5**). Increased abundance of *HA2.1* and *AHA10.1* encoding PM H^+^-ATPase in ectomycorrhizas compared to NM roots of *P.* × *canescens* were suggested to lead to higher activities of PM H^+^-ATPases (Ma Y. et al., 2014). The highly activated PM H^+^-ATPase, on the one hand maintains a more suitable acidic environment to promote the Cd^2+^ and Ca^2+^ influx across the PM (**Figure 10**) and on the other hand, provides an electrochemical H^+^ gradient for PM hyperpolarization, thus increasing Cd^2+^ influx via HACCs. Accordingly, the Cd^2+^-stimulated Cd^2+^ and Ca^2+^ in the *P. involutus* mycelia (**Figures 1**–**3**) was associated with the activated H^+^ pumps since Cd^2+^ treatment markedly upregulated the transcription of PM H^+^-ATPase 1 (Jacob et al., 2004).

Importantly, the H_2_O_2_ produced in the ectomycorrhizal associations may accelerate the Cd^2+^ through the PM H^+^-ATPase-mediated HACCs. Whole-cell patch clamp recordings of *Arabidopsis* guard cells showed that the PM hyperpolarization only activates Ca^2+^ currents in the presence of H_2_O_2_ (50 μM to 5 mM), and the Ca^2+^ current amplitudes increase with increasing H_2_O_2_ concentrations (Pei et al., 2000). Demidchik et al. (2007) showed that application of H_2_O_2_ (10 mM) to the external PM face of elongation zone epidermal protoplasts resulted in the appearance of a hyperpolarization-activated Ca^2+^ permeable conductance. In mature epidermal protoplasts, PM HACCs were activated only when H_2_O_2_ was present at the intracellular membrane face, and channel opening probability increased with intracellular H_2_O_2_ concentrations at hyperpolarized voltages (Demidchik et al., 2007). A massive presence of H_2_O_2_ was demonstrated in the outer hyphae mantle of *P. involutus* symbiosis (Gafur et al., 2004; Langenfeld-Heyser et al., 2007) and obviously could be released from the hyphae into the surrounding medium (**Figure 6**). Therefore, we suppose that in ectomycorrhizal *P.* × *canescens*, H_2_O_2_ elicited by fungal colonization stimulated Cd^2+^ influx through the HACCs that had been activated by *P. involutus* colonization. In addition, we found that Cd^2+^ influx in NAU-roots was less restricted than in MAJ-roots by DMTU and sodium orthvanadate (**Figures 9B** and **11B**). The difference in the sensitivity to antagonists of H_2_O_2_ and PM H^+^-ATPase indicates the involvement of voltage-independent Ca^2+^ channels (VICCs) in the mediation of Cd^2+^ uptake in NAU-roots, in addition to the dominant Cd^2+^ entry through HACCs.

We noticed that LT stress in hydroponic conditions caused a pronounced shift of H^+^ efflux toward an influx in EM roots (**Figure 5**). LT-stressed *P. involutus* mycelia exhibited a trend similar to that in EM roots (**Figure 5**). These results imply that ectomycorrhization activated an H^+^/Cd^2+^ antiport to reduce excessive Cd^2+^ uptake and accumulation under prolonged stress conditions (Sun et al., 2013b). Similarly, we have previously shown that NaCl-treated *P. euphratica* roots retain an active PM Na^+^/H^+^ antiport to avoid the excessive buildup of Na^+^ when exposed to LT salinity (Sun et al., 2009a,b). Here, the rate of H^+^/Cd^2+^ antiport could not be determined, because our NMT data only show the net flux of the target element across the PM, instead of an unidirectional flux. In addition, EM roots were able to avoid the ROS burst in Cd^2+^ environments (**Figure 6**), probably because these roots were characterized by elevated H_2_O_2_ production (Gafur et al., 2004). Therefore, EM roots are likely to control the Cd^2+^ influx through the H_2_O_2_-activated PM Ca^2+^ channels, thus avoiding an excessive accumulation of the heavy metal ions under prolonged period of Cd^2+^ stress.

## Conclusion

**FIGURE 12:**
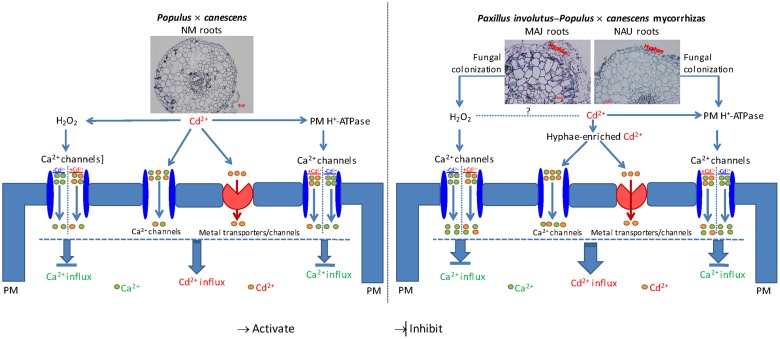
**Schematic models showing Cd^2+^ influx through plasma membrane (PM) Ca^2+^ channels that stimulated by H_2_O_2_ and H^+^-ATPase in *Paxillus involutus*-ectomycorrhizal (MAJ and NAU) and non-mycorrhizal (NM) *Populus* × *canescens* roots under Cd^2+^ stress.** High external Cd^2+^ facilitates the rapid movement of Cd^2+^ along its electrochemical gradient into fungal and plant cells. Cd^2+^ ions penetrated the ectomycorrhizal fungal hyphae and poplar roots through PM Ca^2+^ channels and other metal transporters or channels. The PM Ca^2+^ channels mediate the entry of Ca^2+^ in the absence of Cd^2+^ (-Cd) while allow the entry of Cd^2+^ in the presence of Cd^2+^ ions (+Cd). The Cd^2+^-permeable Ca^2+^ channels were activated by H_2_O_2_ and H^+^-pumping activity. Thus the Cd^2+^-elicited H_2_O_2_ and active H^+^-pumps favored the Cd^2+^ influx through Ca^2+^ channels in NM roots and *P. involutus*-ectomycorrhizas. In ectomycorrhizas, Cd^2+^ enriched in hyphae is thought to be delivered to the host roots. Moreover, the colonization of *P.* × *canescens* roots with the fungal strains MAJ and NAU stimulates H_2_O_2_ production and increases H^+^-pumping activity, and thus accelerates Cd^2+^ entry through Ca^2+^ channels under excessive Cd^2+^. Cd^2+^ ions competitively enter Ca^2+^ channels, and thus diminish the entry of Ca^2+^, leading to a marked Cd^2+^ enrichment in ectomycorrhizal roots under Cd^2+^ stress.

High external Cd^2+^ facilitates the rapid movement of Cd^2+^ along its electrochemical gradient into fungal and plant cells. Based on pharmacological evidence, we conclude that Cd^2+^ ions mainly penetrated the ectomycorrhizal fungal hyphae and poplar roots through PM Ca^2+^ channels. Because the entry of Cd^2+^ could not be fully blocked by various Ca^2+^ channel inhibitors (LaCl_3_, GdCl_3_, verapamil, and TEA), our results indicate that Cd^2+^ ions also entered the root and fungal cells through other metal transporters or channels. Our flux measurements show that the Cd^2+^-permeable Ca^2+^ channels were activated by H_2_O_2_ and H^+^-pumping activity. Altogether based on the current and literature data, we propose a signaling pathway that triggers Ca^2+^-channel-mediated Cd^2+^ influx in NM *P.* × *canescens* roots and explains the pronounced Cd^2+^ stimulation in ectomycorrhizal associations under Cd^2+^ stress. As shown in **Figure 12**, the Cd^2+^-elicited H_2_O_2_ and active H^+^-pumps favored the Cd^2+^ influx through Ca^2+^ channels in NM roots and *P. involutus*-ectomycorrhiza, while these channels mediate Ca^2+^ influx in the absence of Cd^2+^ stress. In ectomycorrhizas, Cd^2+^ enriched in hyphae is thought to be delivered to the host roots. Moreover, the colonization of *P.* × *canescens* roots with the fungal strains MAJ and NAU stimulates H_2_O_2_ production and increases H^+^-pumping activity, and thus accelerates Cd^2+^ entry through Ca^2+^ channels, in particular through HACCs, under excessive Cd^2+^. Cd^2+^ ions competitively enter Ca^2+^ channels, and thus diminish the entry of Ca^2+^, leading to a marked Cd^2+^ enrichment in ectomycorrhizal roots under Cd^2+^ stress.

## Author Contributions

YhZ and SC conceived the original screening and research plans; SC supervised the experiments; YhZ, GS, YnZ, ZZ, and NL performed most of the experiments; SD, JS, JL, JY, NZ, RZ, and XM provided technical assistance to YhZ, GS and YnZ; YhZ designed the experiments and analyzed the data; YhZ conceived the project and wrote the article with contributions of all the authors; SC and AP supervised and complemented the writing. All authors have read and approved the manuscript.

## Conflict of Interest Statement

The authors declare that the research was conducted in the absence of any commercial or financial relationships that could be construed as a potential conflict of interest.

## Footnotes

1 http://www.xuyue.net/
