# Supplementary material for: Paxillus involutus-Facilitated Cd2+ Influx through Plasma Membrane Ca2+-Permeable Channels Is Stimulated by H2O2 and H+-ATPase in Ectomycorrhizal Populus × canescens under Cadmium Stress
Source: Front Plant Sci. 2017 Jan 6;7:1975. doi: 10.3389/fpls.2016.01975 (PMC5216326; doi:10.3389/fpls.2016.01975)
Supplement: Supplementary file 1 [file Table_1.pdf]

**Supplemental TABLE S1. Effects of $Ca^{2+}$ concentrations on sensitivity of $Cd^{2+}$-selective microelectrodes.**

| | $-Ca^{2+}$ | $+Ca^{2+}$ | | | | | | | | $+K^{+}+Ca^{2+}+Mg^{2+}$ |
|---|---|---|---|---|---|---|---|---|---|---|
| Unit: mM | $Cd^{2+}$ (0.01, 0.05, 0.1) | 0.01 | 0.025 | 0.05 | 0.1 | 0.2 | 0.5 | 1.0 | 2.0 | $K^{+}$ (0.1)/$Ca^{2+}$ (0.05)/$Mg^{2+}$ (0.1) |
| Nernst Slope | 34.0476 | 25.1954 | 26.4443 | 28.9053 | 28.4665 | 28.6798 | 27.1430 | 26.9611 | 27.0076 | 29.0247 |
| | ±0.5584 | ±0.4619 | ±0.2704 | ±0.3568 | ±0.5725 | ±0.8838 | ±0.3648 | ±0.3496 | ±0.4301 | ±0.9358 |
| Nernst Intercept | 247.4408 | 239.5143 | 239.6579 | 241.8781 | 237.6448 | 238.7986 | 227.3050 | 221.8072 | 216.1608 | 243.1562 |
| | ±1.4602 | ±1.5502 | ±1.4003 | ±0.9108 | ±1.5195 | ±1.8944 | ±1.2770 | ±1.0566 | ±0.9820 | ±1.2556 |

$Cd^{2+}$-selective microelectrodes were calibrated in $Cd^{2+}$ solutions (0.01, 0.05, 0.1 mM) supplemented with or without 0, 0.01, 0.025, 0.05, 0.1, 0.2, 0.5, 1.0, and 2.0 mM $Ca^{2+}$. In addition, the Nernst slope and intercept of the $Cd^{2+}$ electrodes was calibrated in the measuring solution containing 0.1 mM KCl, 0.1 mM $MgCl_2$, and 0.05 mM $CaCl_2$.
