# Supplementary material for: Paxillus involutus-Facilitated Cd2+ Influx through Plasma Membrane Ca2+-Permeable Channels Is Stimulated by H2O2 and H+-ATPase in Ectomycorrhizal Populus × canescens under Cadmium Stress
Source: Front Plant Sci. 2017 Jan 6;7:1975. doi: 10.3389/fpls.2016.01975 (PMC5216326; doi:10.3389/fpls.2016.01975)
Supplement: Supplementary file 2 [file Image_1.pdf]

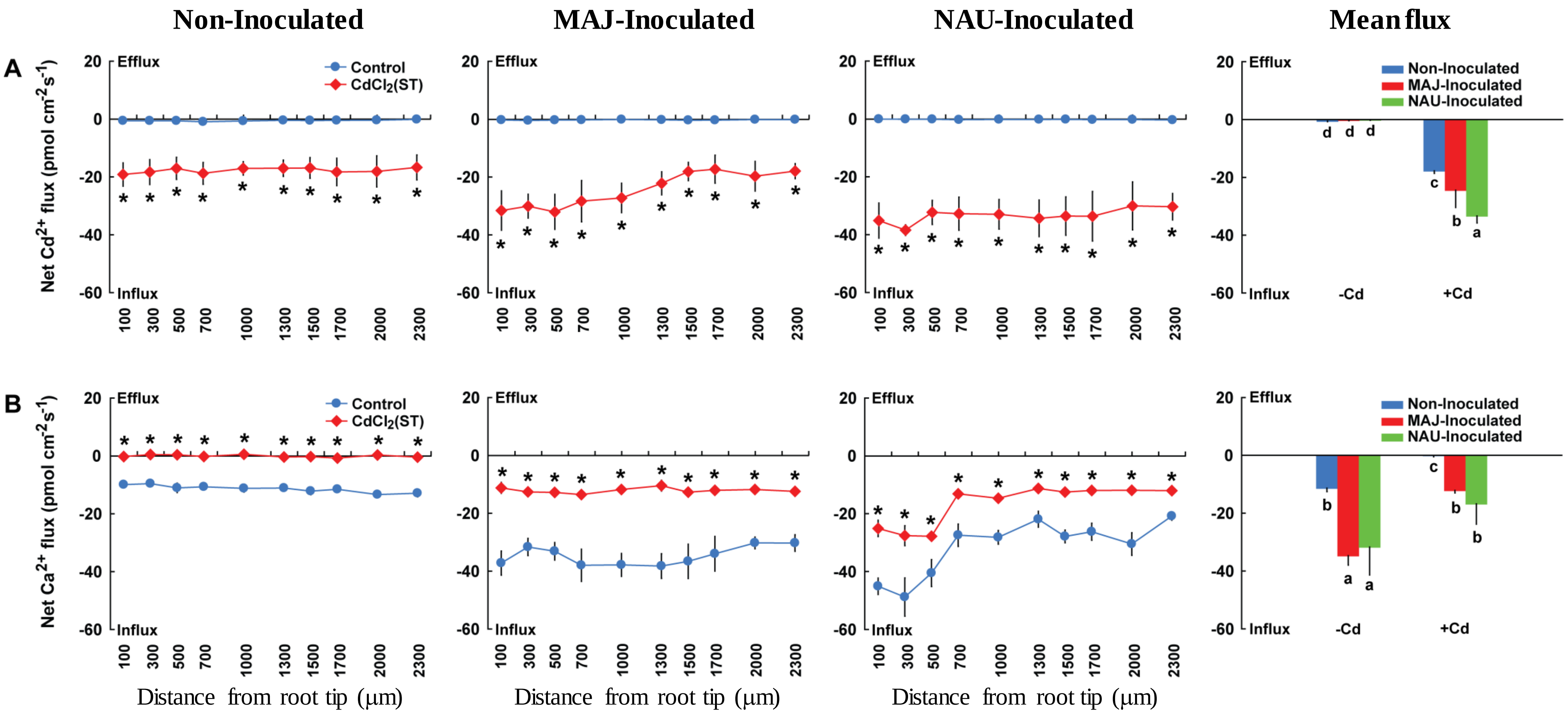

Supplemental FIGURE S1. Effects of $CdCl_2$ on steady Cd^{2+} and Ca^{2+} fluxes in *Populus* × *canescens* roots inoculated with or without *Paxillus involutus* strains. *P.* × *canescens* roots were cultured without or with the *P. involutus* strains MAJ and NAU for 7 days, respectively. Fungal-inoculated and non-inoculated *P.* × *canescens* plants were subjected to short-term (ST, 24 h) exposure to 50 μM $CdCl_2$. Control roots were well fertilized but treated without $CdCl_2$. Cd^{2+} (A) and Ca^{2+} (B) fluxes were measured along root axis, 100–2,300 μm from the apex, at intervals of 200 to 300 μm. Each point is the mean of four to five individual

plants, and bars represent the standard error of the mean. Asterisks denote significant difference at $P < 0.05$ between treatments. The mean values of Cd^{2+} and Ca^{2+} flux before (−Cd) and after (+Cd) the addition of $CdCl_2$ are shown in the right panel. Columns represent the mean of four to five individual plants and bars represent the standard error of the mean. Different letters, a, b, c and d, indicate significant difference at $P < 0.05$ between treatments.

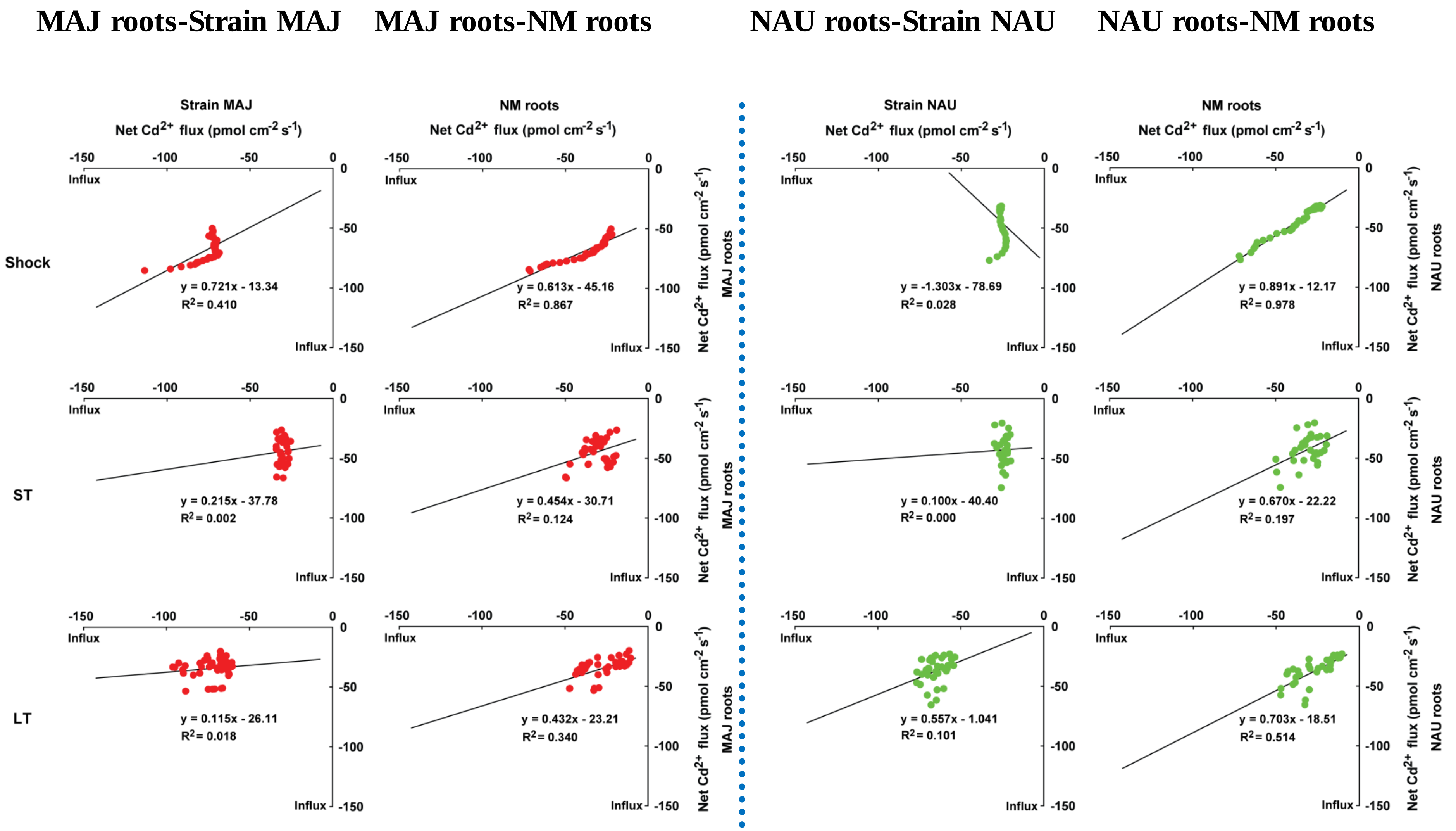

Supplemental FIGURE S2. The correlations of Cd^{2+} fluxes between ectomycorrhizal *Populus* × *canescens* roots, *Paxillus involutus* strains, and non-mycorrhizal (NM) roots under Cd^{2+} stress. *P.* × *canescens* roots (NM, MAJ, and NAU) and *P. involutus* isolates MAJ and NAU were subjected to $CdCl_2$

(50 μM) shock, short-term (ST, 24 h), and long-term (LT, 7 d) treatments, respectively. For shock treatment, transient kinetics of Cd^{2+} in roots (100 μm from the root tip) and axenic mycelia were recorded for a period of 40 min after the required amount of 50 μM $CdCl_2$ was introduced into the measuring solution. The correlations between ectomycorrhizal (EM) roots, NM roots, and *P. involutus* strains were established in terms of measuring time after the flux recordings were started. Under ST and LT treatments, steady fluxes of Cd^{2+} in *P.* × *canescens* roots (NM, MAJ, NAU) were measured along root axis, 100–2,300 μm from the apex, at intervals of 200 to 300 μm. Cd^{2+} fluxes of *P. involutus* isolates MAJ and NAU were measured around the surface of pelleted hyphae over a recording period of 30 min. Each point is the mean of four to five individual plants or axenic EM cultures (pelleted hyphae). The correlations between EM roots and NM roots were established in terms of measuring sites; the correlations between EM roots and *P. involutus* strains were established in terms of measuring time after the flux recordings were started.

MAJ roots-Strain MAJ **MAJ roots-NM roots** **NAU roots-Strain NAU** **NAU roots-NM roots**

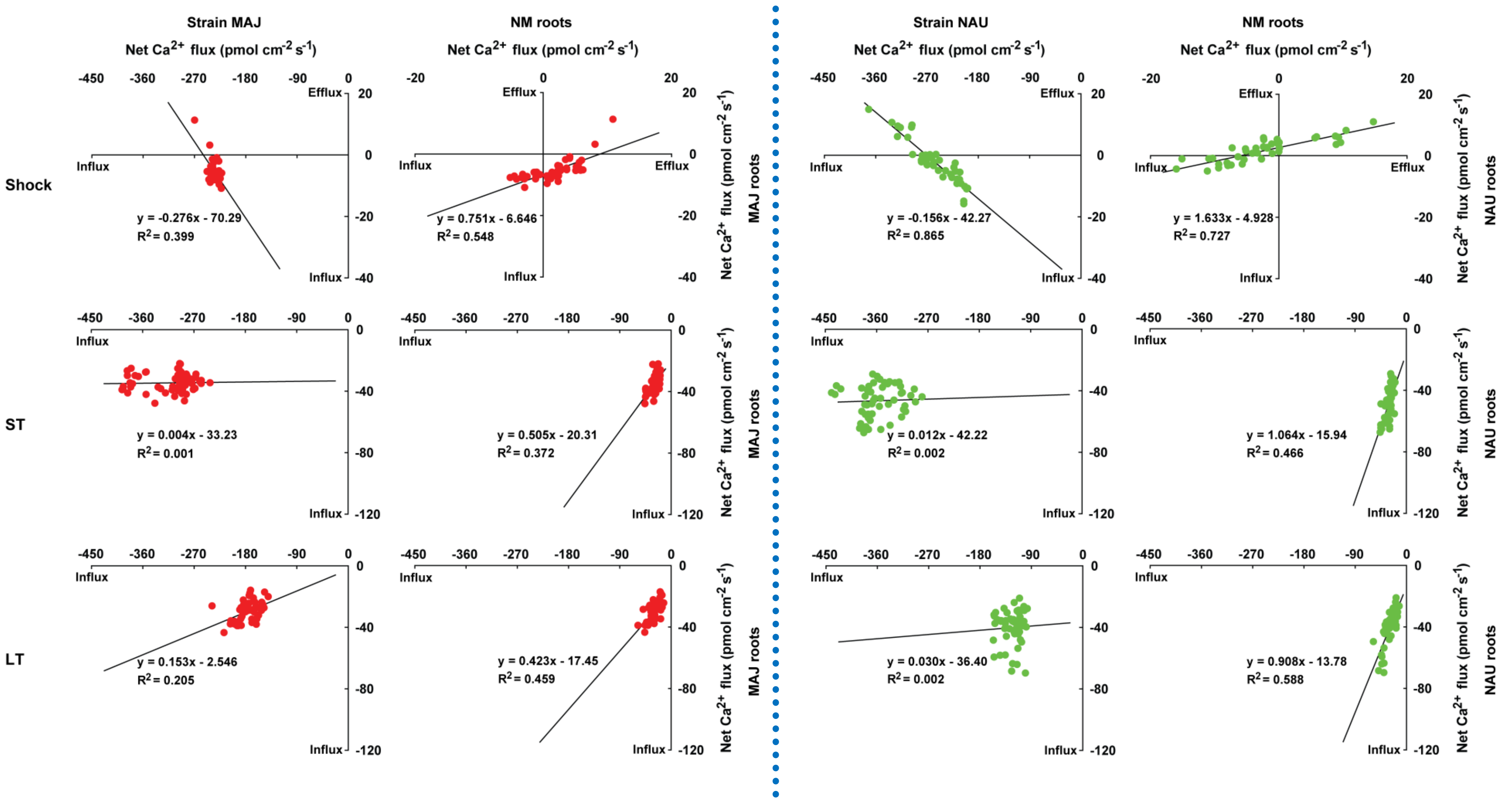

Supplemental FIGURE S3. The correlations of Ca^{2+} fluxes between ectomycorrhizal *Populus* × *canescens* roots, *Paxillus involutus* strains, and non-mycorrhizal (NM) roots under Cd^{2+} stress. *P.* × *canescens* roots (NM, MAJ, and NAU) and *P. involutus* isolates MAJ and NAU were subjected to $CdCl_2$

(50 μM) shock, short-term (ST, 24 h), and long-term (LT, 7 d) treatments, respectively. For shock treatment, transient kinetics of Ca^{2+} in roots (100 μm from the root tip) and axenic mycelia were recorded for a period of 40 min after the required amount of 50 μM $CdCl_2$ was introduced into the measuring solution. The correlations between ectomycorrhizal (EM) roots, NM roots, and *P. involutus* strains were established in terms of measuring time after the flux recordings were started. Under ST and LT treatments, steady fluxes of Ca^{2+} in *P.* × *canescens* roots (NM, MAJ, NAU) were measured along root axis, 100–2,300 μm from the apex, at intervals of 200 to 300 μm. Ca^{2+} fluxes of *P. involutus* isolates MAJ and NAU were measured around the surface of pelleted hyphae over a recording period of 30 min. Each point is the mean of four to five individual plants or axenic EM cultures (pelleted hyphae). The correlations between EM roots and NM roots were established in terms of measuring sites; the correlations between EM roots and *P. involutus* strains were established in terms of measuring time after the flux recordings were started.

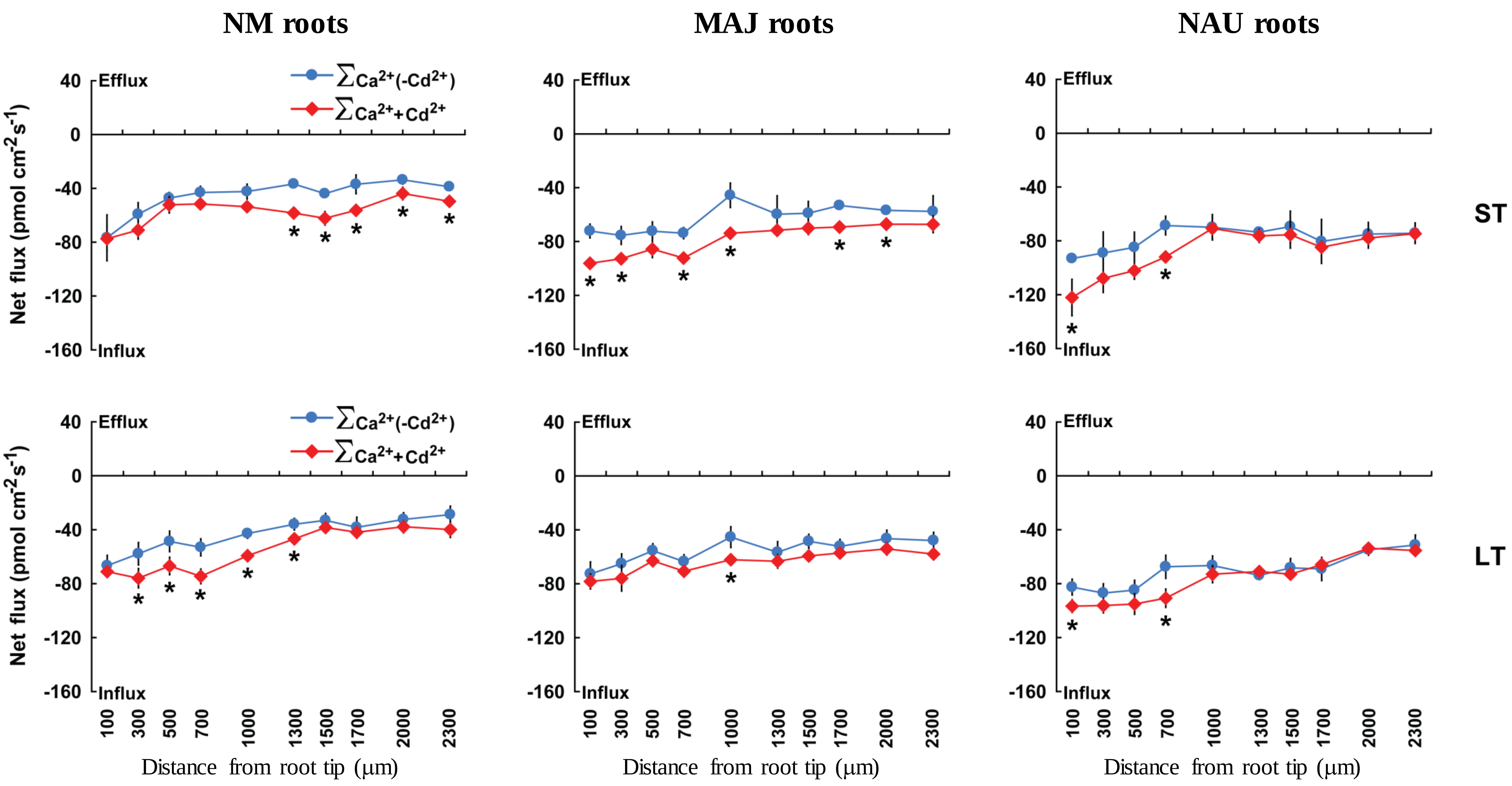

Supplemental FIGURE S4. Total fluxes of Ca^{2+} and Cd^{2+} ($\Sigma_{Ca^{2+}+Cd^{2+}}$) in the presence of $CdCl_2$ (50 µM, $+Cd^{2+}$) and Ca^{2+} flux [$\Sigma_{Ca^{2+}(-Cd^{2+})}$] in the absence of $CdCl_2$ (0 µM, $-Cd^{2+}$) in roots of ectomycorrhizal (MAJ and NAU) and non-mycorrhizal (NM) *Populus* × *canescens*. Ectomycorrhizal (MAJ and NAU) and

NM *P.* × *canescens* plants were subjected to short-term (ST, 24 h) and long-term (LT, 7 d) exposure to 50 μM $CdCl_2$, respectively. Control roots were well fertilized but treated without $CdCl_2$. Ca^{2+} and Cd^{2+} fluxes were measured along root axis, 100–2,300 μm from the apex, at intervals of 200 to 300 μm. Each point is the mean of four to five individual plants, and bars represent the standard error of the mean. Asterisks denote significant difference at $P < 0.05$ between treatments.

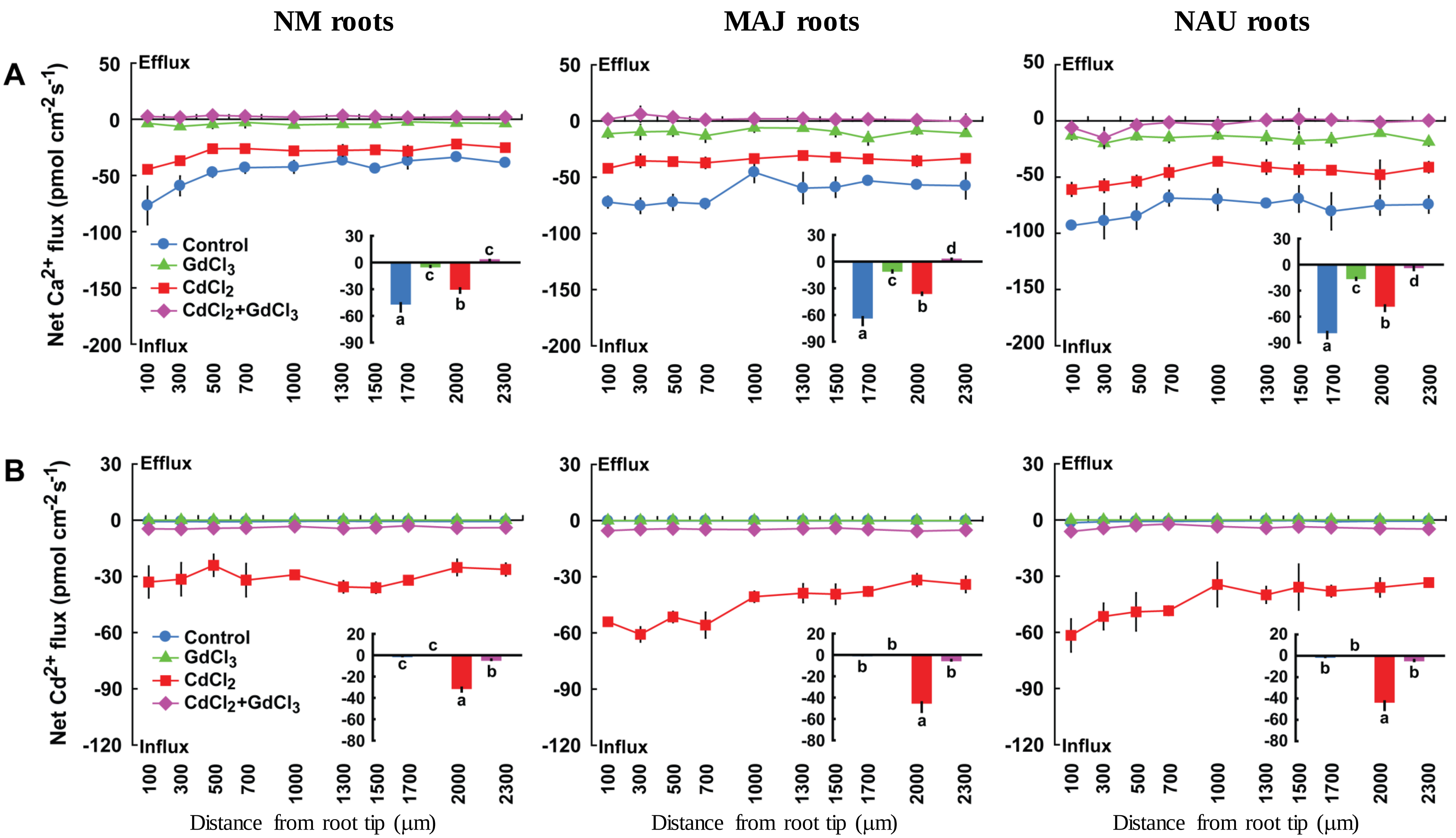

NM roots
MAJ roots
NAU roots
A
B
Net Ca2+ flux (pmol cm-2s-1)
Net Cd2+ flux (pmol cm-2s-1)
Efflux
Influx
Control
GdCl3
CdCl2
CdCl2+GdCl3
Distance from root tip (μm)

Supplemental FIGURE S5. Effects of $GdCl_3$ on steady Ca^{2+} and Cd^{2+} fluxes in roots of ectomycorrhizal (MAJ and NAU) and non-mycorrhizal (NM) *Populus* × *canescens* under Cd^{2+} stress. Ectomycorrhizal (MAJ and NAU) and NM *P.* × *canescens* plants were subjected to 0 or 50 μM $CdCl_2$ for 24 h in the presence and absence of 500 μM $GdCl_3$. Ca^{2+} (A) and Cd^{2+} (B) fluxes were measured along root axes, 100–2,300 μm from the apex, at intervals of 200 to 300 μm. Each point is the mean of four to five individual plants and bars represent the standard error of the mean. Inserted sections show the mean flux rates and different letters, a, b, c, and d, indicate significant difference at $P < 0.05$ between treatments.

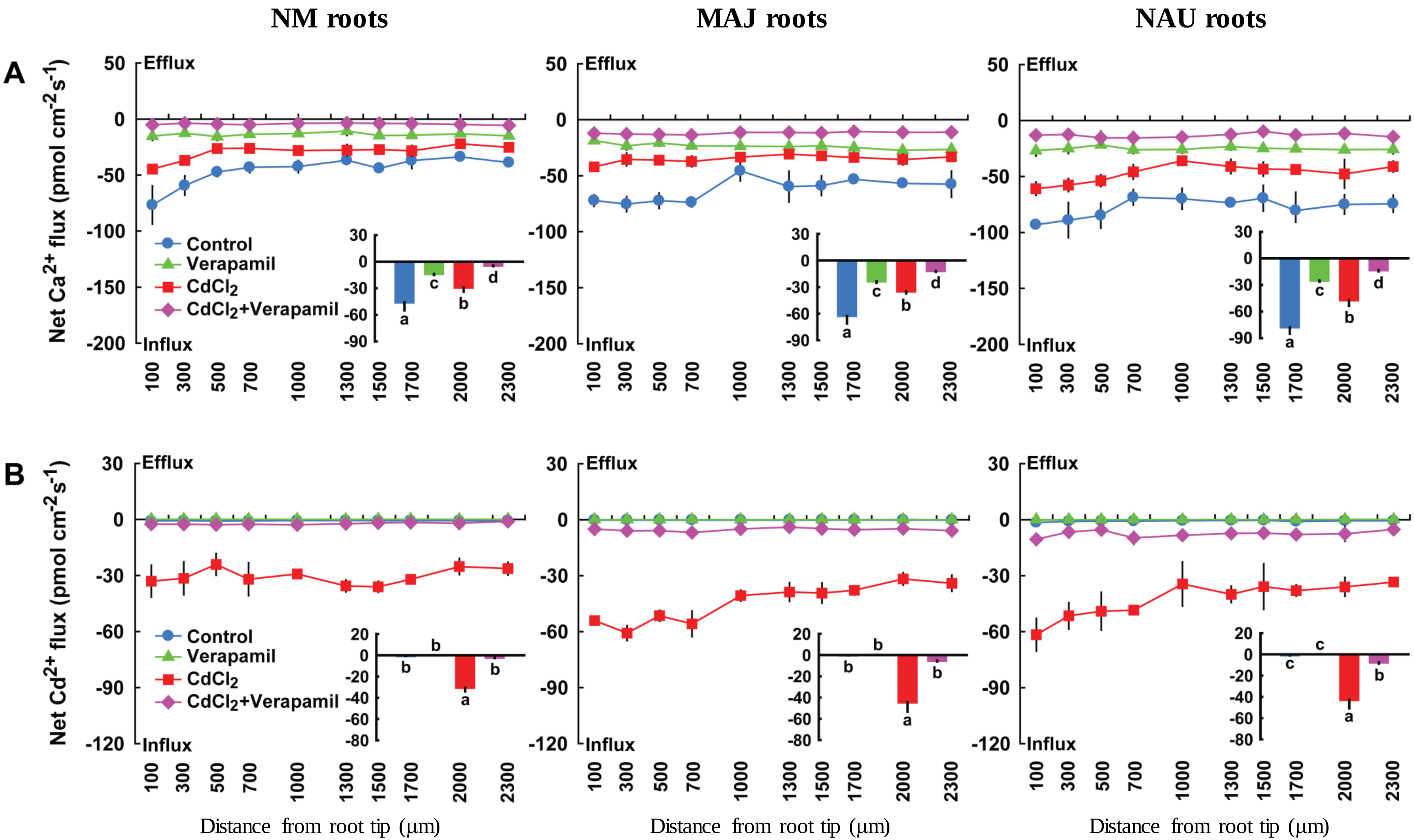

NM roots
MAJ roots
NAU roots
A
B
Net Ca2+ flux (pmol cm-2s-1)
Net Cd2+ flux (pmol cm-2s-1)
Efflux
Influx
Control
Verapamil
CdCl2
CdCl2+Verapamil
Distance from root tip (μm)

Supplemental FIGURE S6. Effects of verapamil on steady Ca^{2+} and Cd^{2+} fluxes in roots of ectomycorrhizal (MAJ and NAU) and non-mycorrhizal (NM) *Populus × canescens* under Cd^{2+} stress. Ectomycorrhizal (MAJ and NAU) and NM *P. × canescens* plants were subjected to 0 or 50 μM $CdCl_2$ for 24 h in the presence and absence of 20 μM verapamil. Ca^{2+} (A) and Cd^{2+} (B) fluxes were measured along root axes, 100–2,300 μm from the apex, at intervals of 200 to 300 μm. Each point is the mean of four to five individual plants and bars represent the standard error of the mean. Inserted sections show the mean flux rates and different letters, a, b, c, and d, indicate significant difference at $P < 0.05$ between treatments.

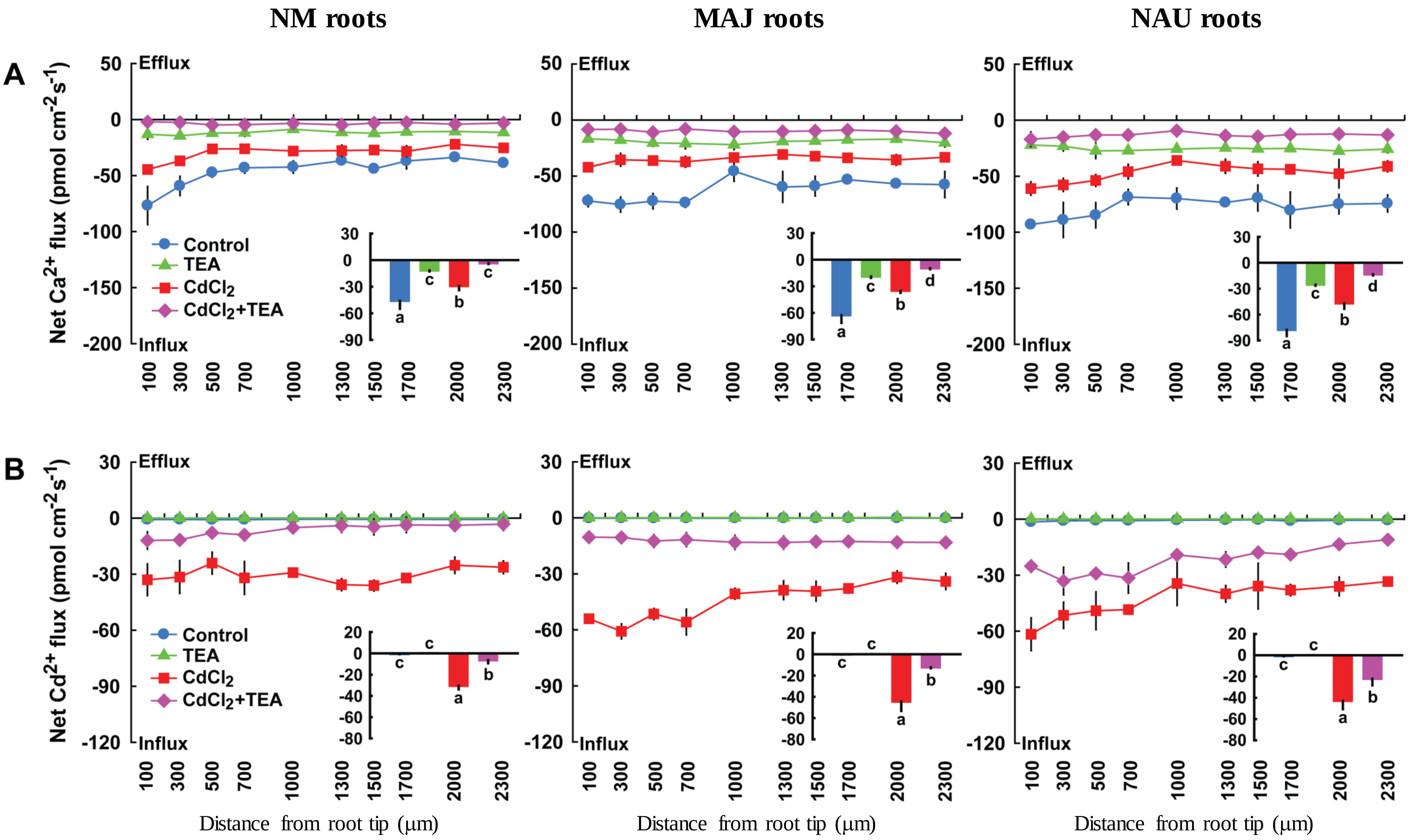
NM roots
MAJ roots
NAU roots
A
B
Efflux
Influx
Net Ca2+ flux (pmol cm-2s-1)
Net Cd2+ flux (pmol cm-2s-1)
Control
TEA
CdCl2
CdCl2+TEA
Distance from root tip (μm)

Supplemental FIGURE S7. Effects of TEA on steady Ca^{2+} and Cd^{2+} fluxes in roots of ectomycorrhizal (MAJ and NAU) and non-mycorrhizal (NM) *Populus × canescens* under Cd^{2+} stress. Ectomycorrhizal (MAJ and NAU) and NM *P. × canescens* plants were subjected to 0 or 50 μM $CdCl_2$ for 24 h in the presence and absence of 50 μM TEA. Ca^{2+} (A) and Cd^{2+} (B) fluxes were measured along root axes, 100–2,300 μm from the apex, at intervals of 200 to 300 μm. Each point is the mean of four to five individual plants and bars represent the standard error of the mean. Inserted sections show the mean flux rates and different letters, a, b, c, and d, indicate significant difference at $P < 0.05$ between treatments.

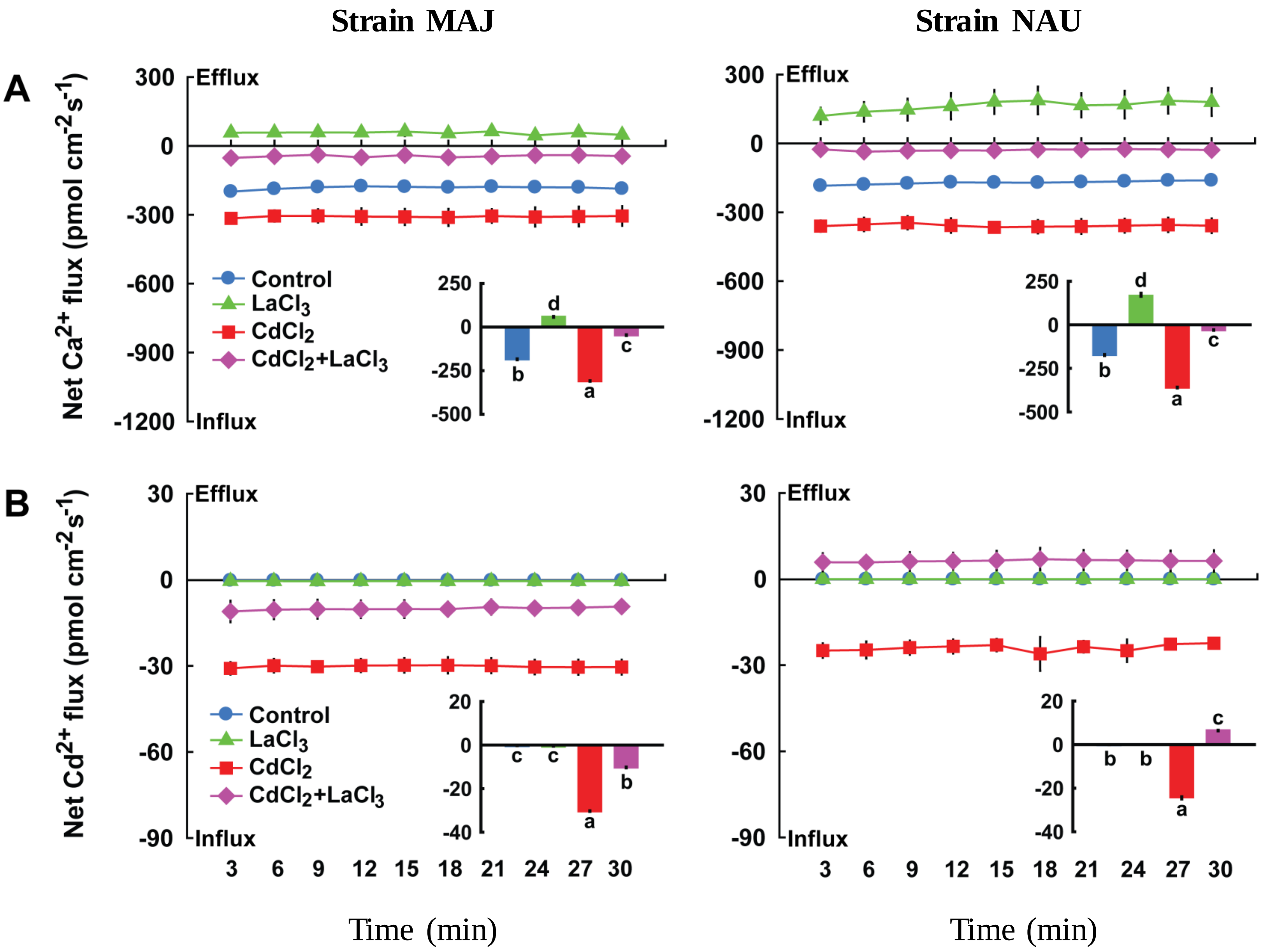

Strain MAJ
Strain NAU
A
B
Net Ca2+ flux (pmol cm-2s-1)
Net Cd2+ flux (pmol cm-2s-1)
Efflux
Influx
300
0
-300
-600
-900
-1200
30
-30
-60
-90
Control
LaCl3
CdCl2
CdCl2+LaCl3
250
-250
-500
20
-20
-40
a
b
c
d
3 6 9 12 15 18 21 24 27 30
Time (min)

Supplemental FIGURE S8. Effects of $LaCl_3$ on steady Ca^{2+} and Cd^{2+} fluxes in *P. involutus* strains MAJ and NAU under Cd^{2+} stress. *P. involutus* isolates MAJ and NAU were subjected to 0 or 50 μM $CdCl_2$ for 24 h in the presence and absence of 5 mM $LaCl_3$. Control axenic mycelia were treated without $CdCl_2$ or $LaCl_3$. Ca^{2+} (A) and Cd^{2+} (B) fluxes were measured along the surface of pelleted hyphae over a recording period of 30 min. Each point is the mean of four to five axenic EM cultures and bars represent the standard error of the mean. Inserted sections show the mean flux rates and different letters, a, b, c and d, indicate significant difference at $P < 0.05$ between treatments.

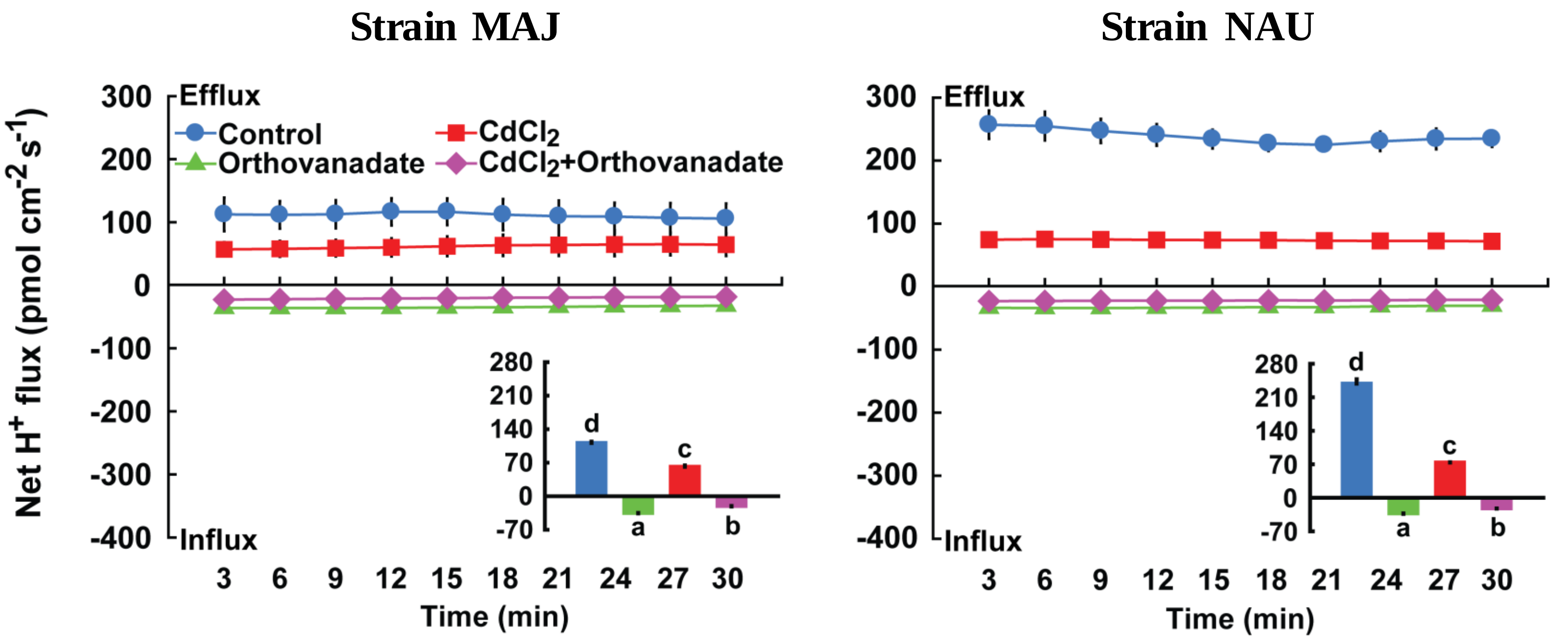

Supplemental FIGURE S9. Effects of sodium orthovanadate on steady H^+ fluxes in *P. involutus* strains MAJ and NAU under Cd^{2+} stress. *P. involutus* isolates MAJ and NAU were subjected to 0 or 50 µM $CdCl_2$ for 24 h in the presence or absence of 500 µM sodium orthovanadate. Control axenic mycelia were treated without $CdCl_2$ or sodium orthovanadate. H^+ flux was measured along the surface of pelleted hyphae over a recording period of 30 min. Each point is the mean of four to five axenic EM cultures and bars represent the standard error of the mean. Inserted sections show the mean flux rates and different letters, a, b, c and d, indicate significant difference at $P < 0.05$ between treatments.
